# Cancer immunotherapies: advances and bottlenecks

**DOI:** 10.3389/fimmu.2023.1212476

**Published:** 2023-08-24

**Authors:** Rui Rui, Liqun Zhou, Shiming He

**Affiliations:** ^1^ Department of Urology, Peking University First Hospital, Beijing, China; ^2^ The Institution of Urology, Peking University, Beijing, China; ^3^ Beijing Key Laboratory of Urogenital Diseases (Male) Molecular Diagnosis and Treatment Center, Beijing, China; ^4^ National Urological Cancer Center, Beijing, China

**Keywords:** cancer immunotherapy, immune checkpoint blockade, tumor microenvironment, neoadjuvant immunotherapy, cancer immunotherapy

## Abstract

Immunotherapy has ushered in a new era in cancer treatment, and cancer immunotherapy continues to be rejuvenated. The clinical goal of cancer immunotherapy is to prime host immune system to provide passive or active immunity against malignant tumors. Tumor infiltrating leukocytes (TILs) play an immunomodulatory role in tumor microenvironment (TME) which is closely related to immune escape of tumor cells, thus influence tumor progress. Several cancer immunotherapies, include immune checkpoint inhibitors (ICIs), cancer vaccine, adoptive cell transfer (ACT), have shown great efficacy and promise. In this review, we will summarize the recent research advances in tumor immunotherapy, including the molecular mechanisms and clinical effects as well as limitations of immunotherapy.

## Introduction

Cancer remains one of the most incurable diseases in the world. Before the 21st century, the main treatments for cancer were surgical resection, radiotherapy, and chemotherapy. Theoretically, cancer can be cured if the tumor tissue is completely removed, however, many cancers have metastasized before they are detected, and most surgical resections are radical removal of the entire organ, causing a certain degree of damage to the patient. Although radiotherapy can kill most of the tumor cells using high doses of radiation, most of the tumor cells will still remain micrometastases, which are difficult to be thoroughly eradicated. Early chemotherapy mainly directly targets on DNA strands, such as platinum compounds and antimetabolites blocking DNA replication and inducing DNA damage. Subsequently derived chemotherapeutic agents center on inhibiting enzymes involved in DNA replication and mitosis, such as camptothecin, anthracyclines, vincristine and paclitaxel. Despite the satisfactory efficacy of chemotherapy, it inevitably damages the patient’s immune system and reduces the quality of life.

More than a century ago, immunological approaches to cancers were first suggested when physicians observed that advanced cancers occasionally faded completely after acute bacterial infections. Immunotherapy has revolutionized cancer treatment and led to a deeper understanding of tumors: treatment of tumors should not only target cancer cells but should take the entire tumor microenvironment (TME) into account. Although the immune system has a complex array of mechanisms to detect and destroy cancer cells, however, tumor cells are deteriorated from autologous epithelial cells and therefore have very low antigenicity and are not easily recognized by the immune system. Although some cancer cells can be recognized by the immune system, some mechanisms inherent in tumor cells help cancer evade the attack of the immune system, which is clearly explained by the concept of cancer immune editing, which emphasizes the dual role of the immune system in suppressing tumor growth while also shaping tumor immunogenicity, describing the process of tumorigenesis using a 3-step process: elimination, equilibrium and escape (1). Immune escape is one of the ten new features of tumors. Although initially cancer cells can be effectively monitored and recognized by the immune system, however, due to the immune-editing effects of cancer, they will eventually enter an immune escape state where the tumor will instead use the immune system to accomplish faster metastasis (2). Tumor immune escape is also one of the bottlenecks in improving the efficacy of current tumor therapy. The mechanism of tumor immune escape is complicated but can be summarized as two aspects: one is the immune escape mediated by tumor cells themselves, such as the absence of major histocompatibility complex (MHC) or co-stimulatory molecules of tumor cells, reduced immunogenicity of tumor antigens, and down-regulation of the expression of genes related to antigen presentation. Second, changes in the function of the body’s immune system, such as the failure of the immune system to recognize low levels of tumor-associated antigens in the early stage of tumorigenesis, the suppression of cellular tolerance and function to tumor-associated antigens caused by myeloid-derived suppressor cells (MDSC), regulatory cells (Treg) and tumor-associated macrophages, and the failure of specialized antigen-presenting cell function (3).

Immune cells are the cellular basis of immunotherapy, and in some highly immune infiltrated tumors, tumor infiltrating leukocytes (TILs) can reach more than 40% (4). Therefore, understanding immune infiltration in TME is key to improving response rates and developing new cancer immunotherapy strategies. Immune cells are the cellular basis of immunotherapy, and in some highly immune infiltrated tumors, tumor infiltrating leukocytes (TILs) can reach more than 40% (4). Therefore, understanding immune infiltration in TME is key to improving response rates and developing new cancer immunotherapy strategies. Although some tumours, such as glioblastoma, have low immune cell infiltration and are referred to as “cold” tumors, there are immunotherapeutic approaches aimed at increasing and activating TILs in the TME of cold tumors to allow more effective antitumor immunity (5).

Immunotherapy, which aims to boost the autoimmune system to remove malignant cells, is a landmark breakthrough in cancer treatment. Despite limited response rates, multiple cancer types have shown sustained clinical responses to immunotherapy (6–8). Several immunotherapies, including immune checkpoint inhibitors (ICIs), cancer vaccine, adoptive cell transfer (ACT), oncolytic virus therapy (OVT), have achieved inspiring results, however, all these treatments in the clinical practice have their own limitations.

## Immune checkpoint inhibitors

Within the tumor, effector T cells have reduced cytokine expression and effector capacity and are resistant to reactivation, a state known as “T-cell exhaustion”. Exhausted T cells highly express a variety of inhibitory surface molecules that effectively prevent T cell activation (9), including cytotoxic T lymphocyte antigen 4 (CTLA-4), programmed death 1 (PD-1), lymphocyte activation gene-3 (LAG-3), and T cell immunoglobulin and ITIM domain (TIGIT). These inhibitory surface molecules are defined as immune checkpoints.

Immune checkpoint blockade (ICB), which targets regulatory pathways in T cells by immune checkpoint inhibitors (ICIs) to enhance anti-tumor immune responses, has shown improved survival over conventional cancer therapy for various cancers and provided a new weapon against tumors. Compared to previous therapies which target to inherent properties of cancer cells, ICB leads to more durable anticancer response *via* boosting anti-tumor response of immune system (10). Currently various ICIs have been approved by FDA (**Table 1**), such as ipilimumab and nivolumab. Newly identified immune checkpoints would also be summarized in this section.

**Table 1:** Summarized immune checkpoint inhibitors approved by FDA or under clinical trials.

Years approved	Target	Drugs	Features	Indication	Response data	Original manufacturer	Reference
2011	CTLA-4	Ipilimumab	fully humanized IgG1κ mAb	Melanoma	ORR 10.9%	Bristol-Myers Squibb^®^	(11)
2014	PD-1	Nivolumab	fully humanized IgG4 mAb	Melanoma	ORR 31.7%	Bristol-Myers Squibb^®^	(12)
				NSCLC	ORR 14.5%		(13)
				RCC	ORR 25%		(14)
				classical Hodgkin’s lymphoma	ORR 69%		(15)
2014	PD-1	Pembrolizumab	fully humanized IgG4 mAb	Melanoma	ORR 33.7%	MerckSharp&Dohme^®^	(16)
				NSCLC	ORR 58%		(17)
				RCC	ORR 59.3%		(18)
				classical Hodgkin’s lymphoma	ORR 69%		(19)
2016	PD-L1	Atezolizumab	FcγR binding-deficient, fully humanized IgG1 mAb	NSCLC	ORR 56%	Roche^®^	(20)
				urothelial carcinoma	ORR 23.5%		(21)
				breast cancer	ORR 53%		(22)
2022	LAG-3	Relatlimab	fully humanized IgG4 mAb	Melanoma	mPFS 10.1 months (with Nivolumab)	Bristol-Myers Squibb^®^	(6)
Not yet approved	TIGIT	Tiragolumab	fully humanized IgG1 mAb with IgG1 backbone effect	NSCLC	mPFS 5.9 months (with atezolizumab)	Roche^®^	(23)
Not yet approved	TIGIT	Vibostolimab	fully humanized IgG1 mAb with IgG1 backbone effec	NSCLC	ORR 26%	MerckSharp&Dohme^®^	(7)

NSCLC, non-small-cell lung cancer; RCC, renal cell carcinoma; ORR, objective response rate, mPFS, median progression-free survival.

## CTLA-4

**Figure 1:**
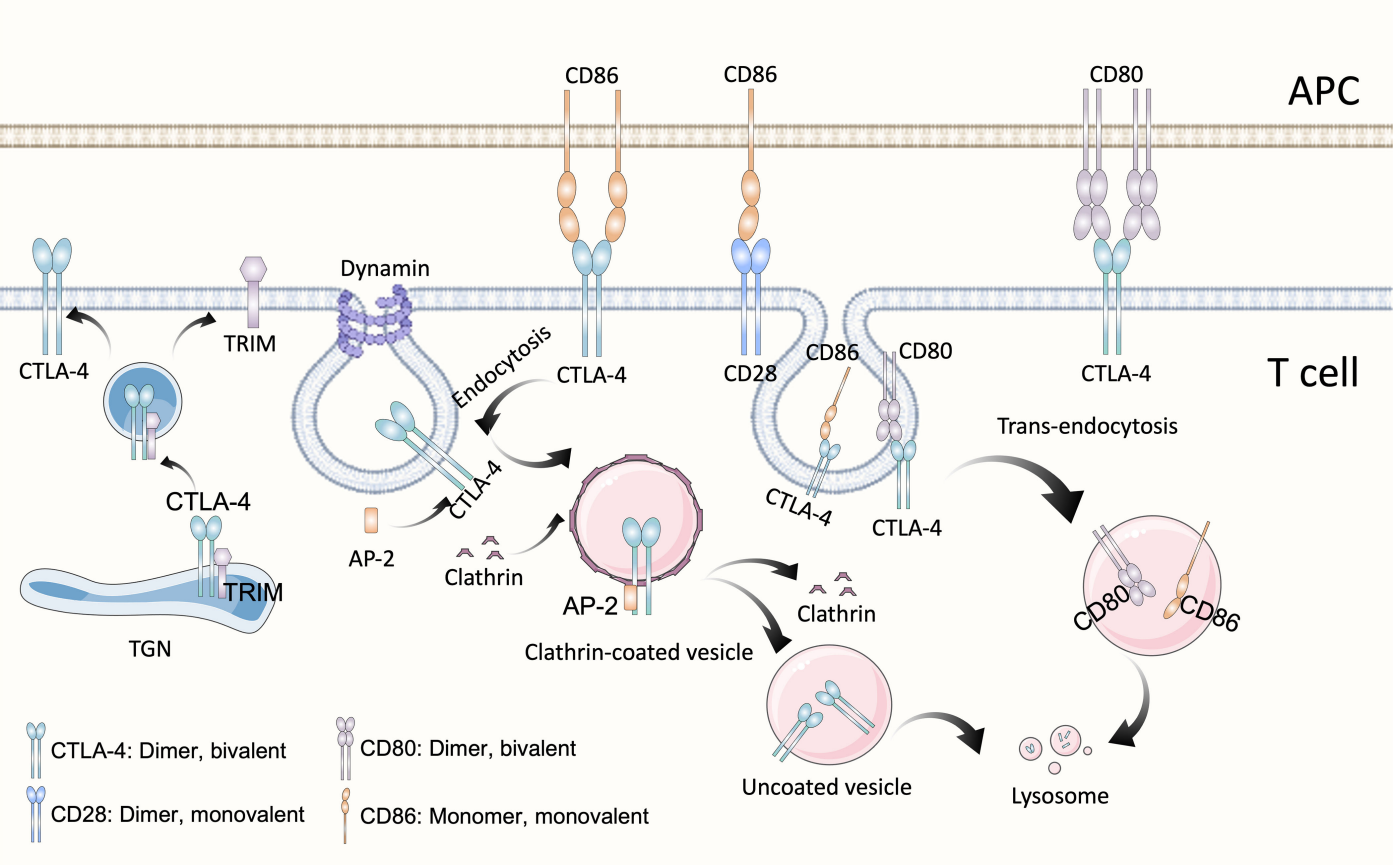
Structure and biological activities of CTLA-4. As homolog of CD28, CTLA-4 possesses the same structure and shares the same ligands B7, CD80 (B7-1) and CD86 (B7-2). CTLA-4 is a divalent dimer, which contains two binding sites, whereas CD28 is a monovalent (which contains a single binding site) dimer. Although both belong to the B7 family, CD80 is a divalent dimer and CD86 is a monomer. CTLA-4 is predominantly present in the intracellular vesicles of T cells, which is due to the constitutive clathrin-dependent endocytosis of CTLA-4 from the plasma membrane, resulting in 90% of CTLA-4 being intracellular. Endocytosis of CTLA-4 is related to the dephosphorylation of its YVKM motif, once CTLA-4 is dephosphorylation, clathrin adapter AP-2 binds to GVYVKM motif of CTLA-4, rapidly inducing internalization. In the TGN, newly synthesized CTLA-4 binds to the transmembrane adapter TRIM, which promotes the formation of CTLA-4-containing vesicles and their transport to the cell surface. CTLA-4, Cytotoxic T-lymphocyte antigen 4; APC, antigen-presentation cell; TGN, trans-Golgi network; TRIM, TCR-interacting molecule; AP-2, μ2 subunit of the clathrin adaptor protein complex.

Cytotoxic T lymphocyte antigen 4 (CTLA-4) (CD152) and CD28 are homologous receptors expressed on the surface of CD4^+^ and CD8^+^ T cells that mediate opposite functions in T cell activation. Recognition of TCR and peptide-MHC class I complexes (pMHC-I) alone is not sufficient to fully activate T cells, activation by co-stimulatory signals is also required, among which the interaction of CD28 and its ligands B7-1 (CD80) and B7-2 (CD86) is most important. The intracellular domain of CD28 contains the YMNM motif and PYAP motif, which can bind to adaptor proteins and several kinases. Some proteins bind to either or both motifs though SH2 or SH3 domain interactions, thereby stimulate IL2 transcription which is mediated by CD28-dependent activation of nuclear factor of activated T cells (NFAT), activator protein 1 (AP-1) and nuclear factor-κB (NF-κB) family transcription factors (24). However, CTLA-4 has much higher affinity and avidity for both CD80 and CD86, binds to CD80/86 and activates intracellular signals that ultimately cause IL-2 downregulation, apoptosis, and anergy (25). CTLA-4 is bivalent homodimer while CD28 is monovalent homodimer, by the way, CD80 is also homodimer while CD86 is monomer, and it has been proved that CD80 may be a more efficient ligand for CTLA-4 based on its bivalent nature (**Figure 1**), whereas CD86 mainly binds to CD28 (26). CD28 is constitutively expressed at the plasma membrane and presents the second signal, but CTLA-4 is predominantly present in the intracellular vesicles of FoxP3^+^ Treg cells or activated T cells, which is due to the constitutive clathrin-dependent endocytosis of CTLA-4 from the plasma membrane, resulting in 90% of CTLA-4 being intracellular, but CTLA-4 trafficking is extremely rapid, with 80% of surface CTLA-4 being internalized within 5 minutes (27). CTLA-4 also degrades CD80 and CD86 by “trans-endocytosis” (28), a mechanism first proposed by Pamela J. Kooh in study of Delta and Notch interactions during Drosophila development, is kind of phagocytosis of transmembrane proteins dependent on receptor-ligand interactions, which may be related to the C terminus of CTLA-4 (29). In this way, CTLA-4 could capture its ligands CD80/86, then CD80/86 are degraded by cells inside cells expressing CTLA-4 (28). However, novel research demonstrated that although CTLA-4 targets both CD80 and CD86 for degradation *via* trans-endocytosis, this process has different consequences for CTLA-4 itself. In the presence of CD80, CTLA-4 remains bound to CD80, being ubiquitinated and trafficked through late endosomes and lysosomes. In contrast, in the presence of CD86, CTLA-4 detaches and recycles back to the cell surface in a PH-dependent manner to allow further trans-endocytosis (30). Besides, CTLA-4 may recruit PP2A, which could dephosphorylate the activated CD28 amino/threonine phosphorylation site, leading to its inactivation (31). These properties of CTLA-4 enable it play negative immunoregulatory effects. In 1996, Leach, D. R. et al. found that anti-CTLA-4 treatment could repress the growth of murine colon carcinoma and murine fibrosarcoma (32), and more than a decade later anti-CTLA-4 monoclonal antibody ipilimumab was used in phase II and phase III clinical study of advanced melanoma in patients who had undergone previous treatment and was proved to improve both overall survival(OS) and progression-free survival (PFS) (11, 33), and ipilimumab was the first immune checkpoint inhibitor approved by US Food and Drug Administration (FDA) for advanced melanoma in 2011. Nevertheless, it has been observed that dose-dependent immune-related adverse events (IRAEs) occurred in ipilimumab treatment (33). Furthermore, CD80 and CD86 are expressed on the surface of antigen-presentation cells (APCs) like dendric cells and monocyte-macrophages, while the non-hematologic tumor doesn’t express CD80 or CD86, so blockade of CTLA-4 is thought to stimulate T cell activation in the secondary lymphoid organs where naive T cells are co-stimulated and differentiate into effector or memory T cell, but not in the tumor microenvironment (24). Besides, CTLA-4 is constitutively expressed on CD4^+^ Tregs, later work demonstrated that the CTLA-4 antibody acted, at least partially, through Fcγ receptor (FcγR)-dependent depletion of tumor-infiltrating Treg cells (34). Activated conventional T cells also express CTLA-4, and there is additional evidence shows that respectively blockade of CTLA-4 on either Treg or effector T cells with selective blocking antibodies enhances the anti-tumor immune effect, suggesting that both Treg and effector T cells are relevant targets of anti–CTLA-4 antibodies (35). Yofe, Ido et al. demonstrated that interactions between anti-CTLA-4 antibodies and FcγR contribute to the conversion of TME to a pro-inflammatory state by inducing Treg cell depletion and myeloid cell reprogramming (36). Although the mechanism of immunotherapy with anti-CTLA-4 monoclonal antibody is not fully understood, the available data support the importance of binding to FcγR.

As the first identified immune checkpoint, anti-CTLA-4 treatment indeed makes great progress in neoadjuvant immunotherapy of cancer. However, there are still some treated patients do not respond. With the increasing interest in neoadjuvant (preclinical) immunotherapy, currently some preclinical trials investigating the use of anti-CTLA-4 antibodies in combination with other immune checkpoint inhibitors are in progress to achieve a more potent therapeutic effect. Although the clinical benefit of CTLA-4 blockade can be improved by combination with PD-1 inhibition, IRAEs are still inevitable which suboptimally limit the doses of anti-CTLA-4 mAb that can be used (37). Dovedi, S. J., et al. designed a monovalent bispecific antibody named MEDI5752 targeting PD-1 and CTLA-4. MEDI5752 preferentially targets CTLA-4 on PD-1^+^CTLA-4^+^ double-positive CD4^+^ and CD8^+^ T cell. When compared with a combination of mAbs targeting the PD-1 and CTLA-4 pathways, MEDI5752 can enhance T-cell activity and can preferentially accumulate in the TME in humanized mice, generating effective antitumor immune responses (38). Besides, it was also proved that, compared with parental mAb, MEDI5752 induced a more rapid time- and dose-dependent internalization and degradation of cell-surface PD-1 in CHO PD-1+CTLA4+ (10:1) cells, perhaps on the result of colocalization of CTLA-4 and PD-1 under MEDI5752 treatment, while parental mAbs resulted in no change in colocalization (38).

## PD-1/PD-L1

**Figure 2:**
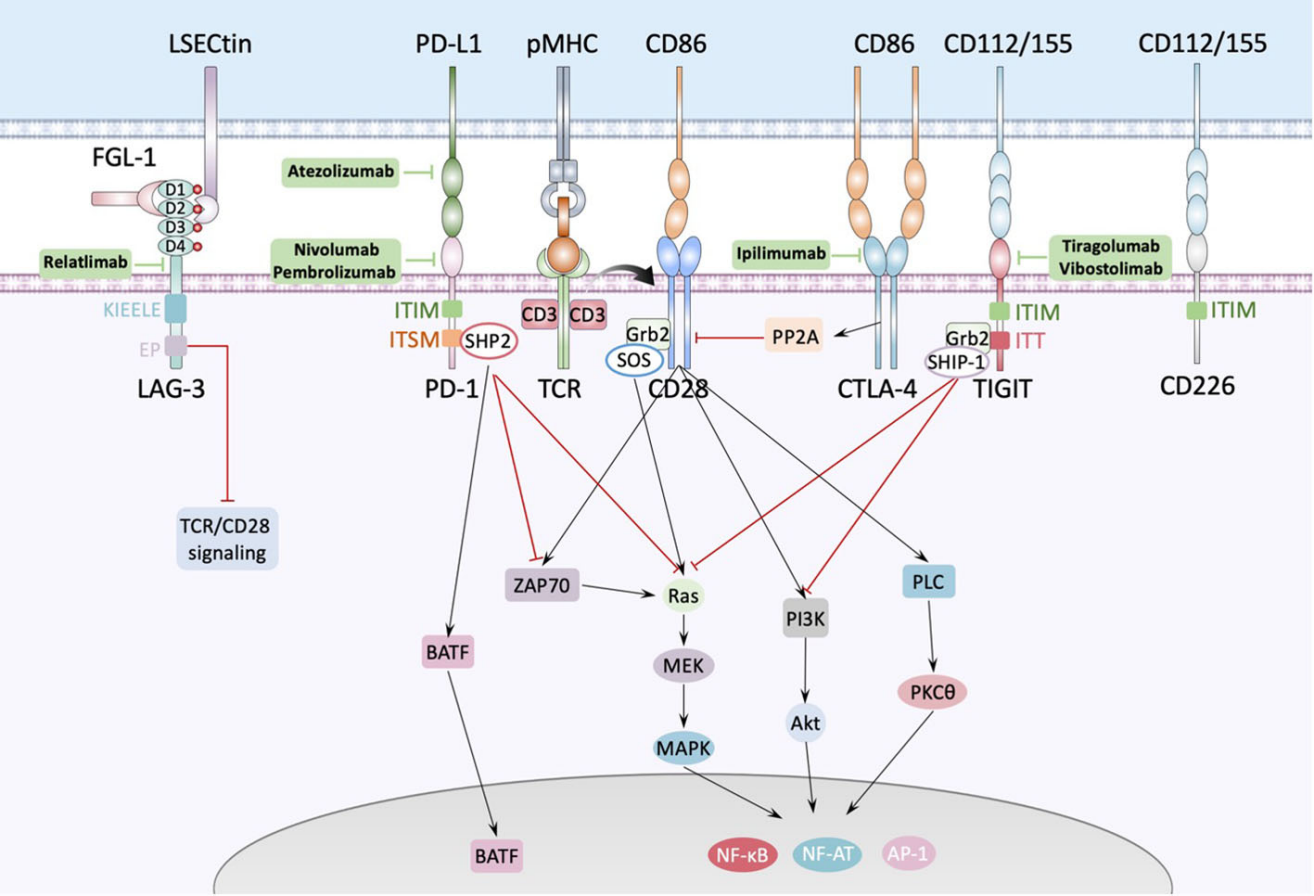
Interactions between antigen presenting cell and T cell. The major histocompatibility complex-peptide (pMHC) complex on APC or tumor cell recognizes and binds TCRs from T cells, while CD80/CD86 binds CD28 and fully activates TCR-CD28 signaling, promoting T cell activation, proliferation, and triggering the expression of associated transcription factors such as nuclear factor of activated T cells (NF-AT), activator protein 1 (AP-1) and nuclear factor-κB (NF-κB). PD1 could bind with PD-L1 expressed by APC or tumor cell and cause dephosphorylation of Zap70 and Ras by recruiting phosphatases, such as SHP2, to the tyrosine-based immunoreceptor switch motif (ITSM) in the intercellular domain of PD1, inducing inhibition of the relevant pathway. Besides, PD1 increases the expression of transcription factors such as BATF (basic leucine zipper transcription factor, ATF-like) to inhibit T cell function. LAG-3 contains four IgSF domains, each of which contains a glycosylation site. There’re several ligands of LAG-3 like LSECtin expressed on the surface of APC or tumor cells, and FGL-1 secreted by tumor cells. FGL-1 binds to D1 and D2 domain of LAG-3 and LSECtin binds to D2 domain of LAG-3. TIGIT is composed of an extracellular Ig variable (IgV) domain, a type 1 transmembrane domain, and a cytoplasmic tail with two inhibitory motifs: an immunoreceptor tyrosine-based inhibitory motif (ITIM) and an Ig tail-tyrosine (ITT)-like motif. Binding to its ligand phosphorylates the cytoplasmic tail of TIGIT which binds to cytosolic adaptor growth factor receptor-bound protein 2 (Grb2), recruiting SH2-containing inositol phosphate-1 (SHIP-1) which inhibits PI3K and MAPK signaling cascades.

The second identified immune checkpoint is programmed death 1 (PD-1, also known as PDCD1 or CD279) pathway. Unlike the rapid regulation of CTLA-4, the regulation of PD-1 depends on transcriptional activation. PD-1 contains a conventional immunoreceptor tyrosine inhibitory motif (ITIM) and an immunoreceptor tyrosine switch motif (ITSM). PD-1’s ITIM and ITSM bind the inhibitory phosphatase SHP-2 (SH2 domain-containing protein tyrosine phosphatase-2) (39). PD-1 engagement directly inhibits TCR-CD28 signaling such as ZAP70, Ras-MAPK and PI3K signaling, as well as increases T cell migration within tissues, thus restraining the time that a T cell has to recognize the surface of interacting cells for the presence of cognate peptide-MHC complexes (**Figure 2**) (39). Besides, PD-l includes upregulation of basic leucine transcription factor, ATF-like (BATF), a transcription factor in the AP-1 family to impair T cell proliferation and cytokine secretion (31). Because its ligands PD-L1 (also known as CD274 or B7-H1) and PD-L2 (also known as CD273 or B7-CD) are mainly expressed on the surface of tumor cells and tumor infiltrating leukocytes (TILs), it has been demonstrated that tumor escaped from immune surveillance by expressing PD-L1/L2, thereby suppressing TILs *via* PD-1/PD-L1,2 interactions, so anti-PD-1 strategy is viewed as predominately play role in TME. Normally, the naive T cells, effector T cells (T_eff_) and memory T cells (T_M_) are defined as PD-1^-^CD8^+^, however, tumor infiltrating CD8^+^T cells which undergo chronic antigen exposure and stimulation of the TCR, would be dysfunctional due to its exhausted state, called exhausted T cells (T_ex_). Chronic infection with the clone 13 strain of LCMV is the gold standard experimental model for studies of T cell dysfunction or exhaustion. In this model, PD-1 expression was shown to correlate strongly with the severity of infection (40). Bulk and single-cell RNA profiles of CD8^+^ TILs revealed that combination of Tim-3 and PD-1 blockade expanded the subset of PD-1^-^CD8^+^ T cells in the TME, and shift CD8^+^ T cells from naive-like to T_eff_ or T_M_, which promoted durable antitumor immune responses and positively correlated with the prognosis of tumor patients (41). The first anti-PD-1 inhibitor nivolumab (BMS-936558 or MDX1106), a fully human IgG4 monoclonal antibody, manufactured by Bristol-Myers Squibb, was approved by FDA in 2014, and proved that be effective in several cancers, like melanoma (42), advanced nonsquamous non-small-cell lung cancer (NSCLC) (43), squamous NSCLC (44), advanced renal cancer carcinoma (14), relapsed or refractory Hodgkin’s lymphoma (45) and so on.

Despite promising progress in anti-PD-1 therapy, most patients didn’t show durable remission, and some cancers have been completely insensitive to response with checkpoint blockade. Combination with other immune checkpoint inhibitors is a suitable approach. In the 4T1.2 breast cancer mouse model of neoadjuvant immunotherapy, triple combination of anti-CTLA4 and anti-PD1and IL-2 cured almost twice as many mice as compared with dual checkpoint inhibitor therapy (8).

## LAG-3

Lymphocyte activation gene-3 (LAG-3), a human gene of the Ig superfamily (IgSF), consisting of three regions: the intracellular, transmembrane, and extracellular regions which contains four immunoglobulin superfamily (IgSF) domains, D1, D2, D3, and D4 (**Figure 2**), which include eight cysteine residues and 4 N-linked glycosylation sites, is upregulated on activated CD4^+^ and CD8^+^ T cells and a subset of natural killer (NK) cells. In addition to effector CD4^+^ T cells, LAG-3 is also expressed on regulatory T cells (Treg). LAG-3 is expressed on both activated natural Treg (nTreg) and induced CD4^+^ FoxP3^+^ Treg (iTreg) to help Treg execute immune inhibitory functions (46). The cytoplasmic tail of LAG3 consists of three conserved motifs: the first is a potential serine phosphorylation site, however no function has been ascribed to this motif thus far, the second is KIEELE motif which is a highly unique and conserved six amino acid sequence and is proved to be essential for interaction with downstream signaling molecules and further inhibit T cell functions (47). The third is EP motif which is also conserved and contains a glutamic acid and proline dipeptide repeat and is also conserved. Iouzalen et al. reported that LAG-3-associated protein (LAP) bound to the EP motif LAG-3 by using yeast two-hybrid cloning experiment (48). However, the exactly immune-inhibitory mechanism of KIEELE and EP motif has not been elucidated. The sequence data and the chromosomal localization revealed that LAG-3 is closely related to CD4 (49). Indeed, LAG-3 is structurally like the CD4 co-receptor and binds to pMHC-II with a higher affinity than CD4. LAG-3 impacts the function of CD8^+^ T cells and NK cells, neither of which interact with pMHC-II, suggesting that there might be alternate ligands for LAG-3. Xu et al. found that LSECtin, a cell surface lectin of the DC-SIGN family expressed in dendritic cell (DC) that inhibits T-cell responses (50). LSECtin is expressed in many tumors as well as normal liver. Although it has been proved that fibrinogen-like protein 1 (FGL1), a liver-secreted protein, is a LAG-3 functional ligand which binds to the extracellular D1 and D2 domain of LAG-3 independent from pMHC-II, exhibiting negative regulation of a T cell activation (51), however, Maruhashi T et al. demonstrated that binding of LAG-3 to stable pMHC-II but not to FGL-1 induced T cell suppression. Consistently, LAG-3 mutants lacking FGL1-binding capacity but not those lacking stable pMHC-II binding capacity retained suppressive activity, suggesting that blocking of pMHCII-LAG-3 interactions is a potential therapeutic target (52). Up to 2021, the first-in-class human IgG4 LAG-3 blocking antibody relatlimab (BMS-986016) was used in a phase 2-3 clinical trial in combination with nivolumab in untreated advanced melanoma, it showed that relatlimab–nivolumab dual checkpoint inhibition had twice the median PFS and a 25% reduction in the risk of disease progression or death compared to nivolumab alone (6). Besides, the frequency of EMOE^+^CD8^+^ T cells in post-treatment samples was higher in responding patients after relatlimab and nivolumab treatment, achieving a 70% pathologic remission rate in patients with resectable stage III or IV hypermetastatic melanoma with a favorable safety profile (53). Nivolumab is a current standard therapy for melanoma. These studies demonstrated the clinical promise of relatlimab. In 2022, Bristol Myers Squibb developed a fixed-dose combination immunotherapy (Nivolumab and relatlimab-rmbw; Opdualag™) for the treatment of multiple types of advanced cancer, which is the first FDA-approved LAG3 monoclonal antibody combination therapy, making LAG3 the third immune checkpoint in clinical use after PD-1 and CTLA-4.

## TIGIT

T cell immunoglobulin and ITIM domain (TIGIT), is a receptor of the Ig superfamily, which plays a critical role in limiting adaptive and innate immunity. It is expressed on NK cells, Treg cells, effector and memory T cells and is also known as Vstm3 (54), WUCAM (55) and TIGIT (56). There are two ligands binding TIGIT: the nectin family CD155 and CD112, which are expressed on APCs, T cells, and a variety of non-hematopoietic tumor cells. Like CTLA-4, TIGIT also has its positive counterpart CD226, which promotes cytotoxicity and enhances anti-tumor responses, executing immunostimulatory functions when binds to CD155 and CD112, however, TIGIT has much higher affinity than CD226 (57). The TIGIT IgV domain and CD155 D1 domain have a typical Ig β-sandwich fold, and two TIGIT/CD155 dimer assemble into a heterotetramer, which mediate cell adhesion and signaling (58). Up to date, the majority of the anti-TIGIT clinical candidates for anti-TIGIT are IgG1 isoforms known to interact with the high-affinity Fcγ receptors (Fcγ R). Others are mutations in the Fc structural domain of IgG1 that enhance or abolish the binding of Fcγ R or have IgG4 isotypes with a limited ability to interact with Fcγ R. Tiragolumab is a fully humanized IgG1 anti-TIGIT monoclonal antibody with IgG1 backbone effect (59). In tiragolumab plus atezolizumab (anti-PD-L1) treatment, median PFS of NSCLC patients was higher (5.4 months versus 3.6 months) than placebo plus atezolizumab treatment (23). However, 14 (21%) patients receiving tiragolumab plus atezolizumab and 12 (18%) patients receiving placebo plus atezolizumab had serious treatment-related adverse events (TRAEs), among which two treatment-related deaths (of pyrexia and infection) occurred in the tiragolumab plus atezolizumab group. Vibostolimab is another humanized anti-TIGIT mAb with an effector IgG1 backbone. In the first-in-human phase I study, vibostolimab in combination with pembrolizumab therapy improved objective response rate (ORR) (37.3% versus 20.6%) and median PFS (5.6 versus 3.9 months) compared with atezolizumab monotherapy with a tolerable safety profile (60).

## CD47

The CD47-SIRPα (signal-regulatory protein-α) axis is the first recognized phagocytosis checkpoint in innate immune cells. The CD47 protein (also known as integrin-associated protein, IAP) is a transmembrane protein expressed on both healthy and cancer cells, transducing a ‘don’t eat me’ signal when it binds to the SIRPα receptor expressed on myeloid cells to negatively regulate phagocytosis. Binding of CD47 and SIRPα leads to phosphorylation of immunoreceptor tyrosine-based inhibition motif (ITIM) on SIRPα and recruitment and activation of Src homology phosphatases 1 (SHP-1) and 2 (SHP-2) (mainly SHP-1 in macrophages), both of which inhibit accumulation of myosin-IIA at the phagocytic synapse, and interrupt signaling from tyrosine kinase-dependent receptors like the M-CSF receptor c-fms, thereby inhibits phagocytosis of macrophages (61, 62), anti-body-dependent cellular cytotoxicity (ADCC) mediated by NK cells (63), and neutrophil-mediated killing of tumor cells (64).

Because of their determination of tumor cell phagocytosis, antibodies that block the CD47-SIRPα axis show great application prospects. However, both anti-CD47 and anti-SIRPα antibodies contain Fc segments, which could which lead to the activation and engagement of FcR on macrophages, leading to phagocytosis. This leads to a debate about CD47-SIRPα blocking agents that whether blocking the interaction between SIRPα and CD47 alone, independent of FcR activation, is sufficient to trigger macrophage phagocytosis and tumor cell elimination (65). Nevertheless, in response to anti-CD47 F(ab’)_2_ fragments treatment, macrophages are much more efficient at engulfment of haematopoietic tumor cells such as human B-cell tumor cell lines Raji and Daudi, compared with non-haematopoietic tumor cells like colon carcinoma cell lines Colo205, SW480, and SW620. Chen, J., et al. proved that phagocytosis of haematopoietic tumor cells during SIRPα–CD47 blockade was strictly dependent on signaling lymphocytic activation molecule (SLAM) family receptors *in vitro* and *in vivo (* 66), indicating that engagement of FcR was not needed for phagocytosis of haematopoietic tumour cells such as Raji cells. But it remains unclear which of the CD47-SIRPα blockers, such as anti-CD47 antibodies, anti-SIRPα antibodies or soluble SIRPα proteins, provides the greatest antitumor efficacy and the least toxicity, and secondly, whether these agents should contain an Fc fraction requires further study.

Although the discovery of ICI ushered in a new era of immunotherapy, revealing the interactions between tumor cells and immune cells in TME and leading to a deeper understanding of the complex TME, there’re still many difficulties in clinical practice: some patients are unresponsive to ICIs or ICIs-refractory, possibly due to lack of infiltration of immune cells in the TME, or low expression of immune checkpoints. The assessment of TILs played a vital role. Assessment of TILs in TME was first performed in colorectal cancer (CRC). By observing the type, density and location of immune cells within the TME, survival in CRC can be predicted more accurately than with the traditional TNM system (67), leading to the development and implementation of the immunoscore, a robust and consistent standardized scoring system based on the quantification of two lymphocyte populations (CD3 and CD8) at the tumor center and invasive margin (68). By classifying cancers according to immune infiltration, the system introduces the four proposed types of tumour based on Immunoscore: hot, altered(excluded, immunosuppressed) and cold. The “hot” tumor is defined as highly T cell-infiltrated with immune score I4 while “cold” tumor is defined as non-infiltrated with immune score I0. There’s the tricky issue that cold tumor scarcely expresses PD-L1 and MHC-I, which makes it immunologically ignorant and intractable and hardly respond to ICI. Therefore, converting “cold” tumors into “hot” tumors in response to ICIs is one of the hot topics in this field. One of recommended approaches is combining therapies enhancing T cell responses with the removal of co-inhibitory signals and/or the supply of co-stimulatory signals to increase functional T cells to response to ICI (69).

Although cancers can be classified as “cold” or “hot” tumors based on the evaluation of TILs, however, due to the heterogeneity of cancer and individual differences, different patients with the same tumor exhibit widely varying response rates to ICIs. Therefore, when selecting the most appropriate therapeutic regimen, robust biomarkers need to be identified to determine which patients are suitable for treatment with ICIs.

## Cancer vaccine

**Figure 3:**
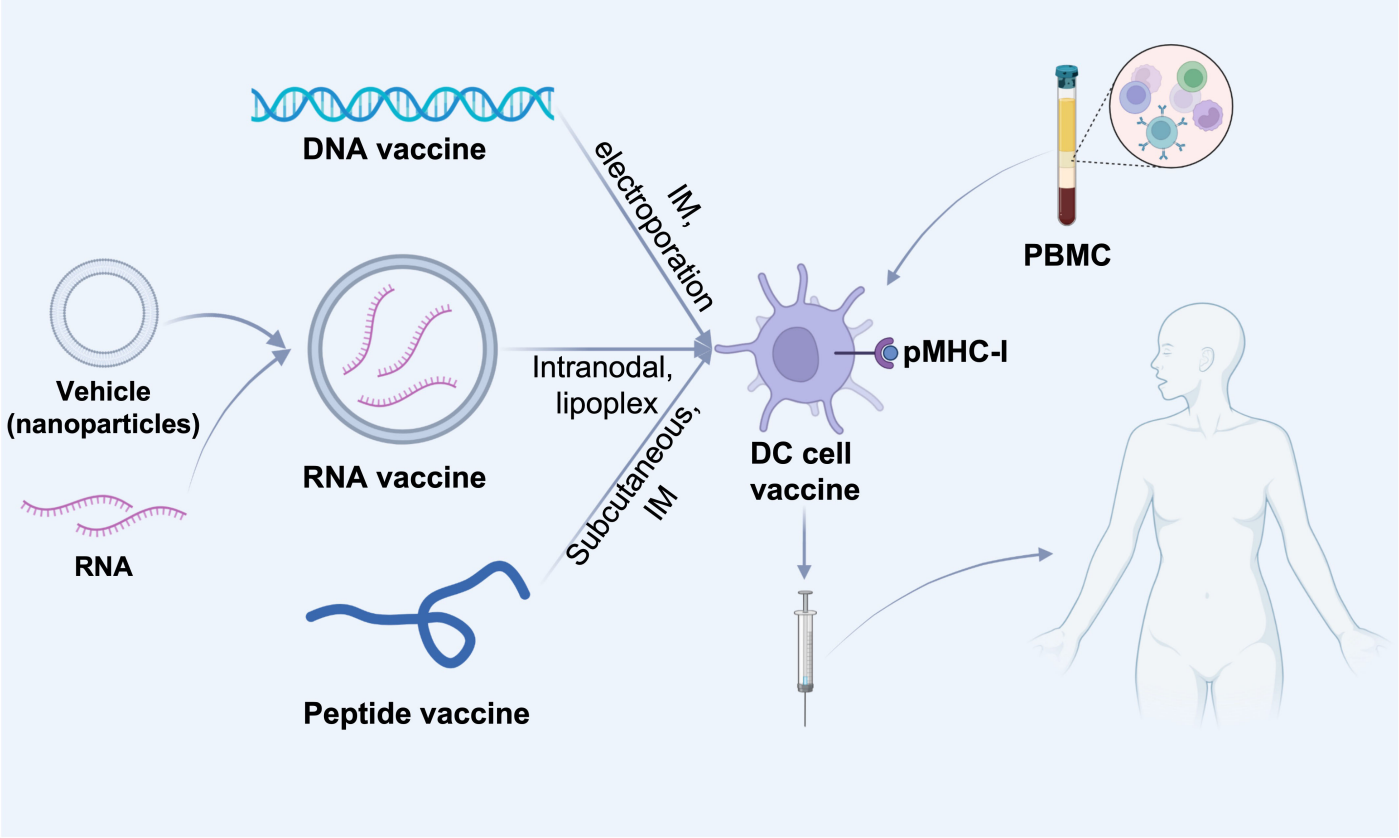
Different cancer vaccine administration routes. DNA or RNA or peptides of screened tumor-associated antigens are injected into dendritic cells in tumor-draining lymph nodes by various routes (intravenous, intramuscular or subcutaneous). DNA or RNA vaccines can be delivered directly intratumorally, e.g., DNA vaccines can be electroporated directly at the injection site, and RNA vaccines can be delivered intravenously *via* nanoparticles (e.g., liposomes), which contribute to the delivery of the vaccine to lymph node-resident dendritic cells (DCs). In addition, DNA and RNA vaccines can also be delivered *in vitro* by presenting to patient-derived PBMC-isolated DC cells, which are then administered subcutaneously, intramuscularly, intranodally, or intravenously into the patient. IM, intramuscular; pMHC-I, peptide-MHC class I complexes; PBMC, Peripheral blood mononuclear cell.

The clinical application of ICIs has greatly facilitated cancer treatment. However, there’re still limitations since tumor cells have intrinsic anti-ICIs mechanism, besides, tumor cells have a high degree of heterogeneity. Therefore, it is important to explore tumor-specific antigens to provide more precise treatment approaches while tumor vaccines can meet these needs. The world’s first preventive tumor vaccine Gardasil for clinical use was approved by the FDA in 2006, for the prevention of uterine cancer caused by HPV16 and HPV18 infections (70). In 2010, PROVENGE (sipuleucel-T), a DC vaccine, the world’s first therapeutic cancer vaccine for clinical use, was approved by the FDA for treatment of patients with advanced prostate cancer, especially those refractory to hormone therapy (71). In fact, since the start of immunotherapy, people have invested numerous passion and efforts in the research of tumor vaccines. There are mainly three types of therapeutic cancer vaccines: cell vaccines, peptide vaccines, and nucleic acid vaccines (**Figure 3**). The role of tumor vaccines is mainly to increase the infiltration of TILs in the TME or to increase the anti-tumor activity of TILs. Although tumor vaccines seem very promising, their ultimate clinical application is very limited.

## Cancer cell vaccines

Cancer cell vaccines mainly contain two types: autologous tumor cell-based vaccine and DC vaccine. An autologous tumor cell-based vaccine is usually irradiated, CpG-activated tumor cells from patient, reserving most C which activates T cells. In a phase III study, infusion with autologous tumor cell–bacillus Calmette-Guérin (BCG) vaccine after surgical resection in stage II and III colon cancer patients didn’t show statistically significant differences in clinical outcomes compared with the arm under surgical resection alone, though there was a slight trend in PFS and OS (72). The autologous tumor cell-based vaccine is also used in renal cancer carcinoma (73, 74), head and neck squamous cell carcinomas (75), B-cell chronic lymphocytic leukemia (76), NSCLC (76), mantle cell lymphoma (77) and so on. In many cases, the tumor cell vaccine is genetically modified to add more functions such as cytokines production and expression of pro-inflammatory molecules because irradiated tumor cells alone are not sufficient to elicit vital anti-tumor immunity. For instance, by transfection with pGEG.mIL-12 and pGEG.mIL-18 plasmids by means of EBV/lipoplex, the engineered B16 melanoma vaccine could secrete approximately 10 times higher level of related cytokines than transfection with pG.mIL-12 and pG.mIL-18, inducing IFN-γ production and cytotoxic T lymphocyte (CTL) and natural killer (NK) activation, in mice B16 melanoma model, this engineered vaccine showed strong tumor suppression (78). Among many cytokines, irradiated B16 melanoma tumor cells expressing granulocyte-macrophage colony-stimulating factor (GM-CSF) could arouse potent, durable, and specific anti-tumor immunity, activating both CD4^+^ and CD8^+^ cells. That was how GVAX (GM-CSF transduced tumor cell vaccine) produced (79). Despite its effective immune modulatory which has generated research enthusiasm, its clinical effect is not satisfactory. A phase II study showed that maintenance therapy with GVAX and ipilimumab did not improve OS with continuous chemotherapy, but instead resulted in lower survival values for metastatic pancreatic ductal adenocarcinoma (80).

The other tumor cell vaccine is based on dendritic cells (DC). DC have an important role in mediating innate immune responses and inducing adaptive immune responses and have been recognized as the most potent antigen presenting cells (APC), activating both naive and memory immunity. One possible approach is to use DC which present tumor neoantigens which are highly patients specific. Sipuleucel-T is a therapeutic vaccine based on DC in advanced prostate cancer. In this approach, autologous peripheral-blood mononuclear cells (PBMC) are separated which are enriched DC, incubating with a recombinant fusion protein (PA2024) consisting of a prostate antigen, prostatic acid phosphatase, and GM-CSF. In metastatic castration-resistant prostate cancer, there was a relative reduction of 22% in the risk of death with a 4.1-month improvement in median survival (25.8 months versus 21.7 months) in Sipuleucel-T treated group compared with placebo group (71). DC-based cancer cell vaccines therefore show great potential and application prospects and have been used to melanoma (81), lung cancer (81),and other solid tumors, testifying application of DC in tumor is safe and feasible.

## Cancer peptide vaccine

Tumor peptide vaccines mainly based on HLA-restricted antigen epitope which could induce both CD4^+^ and CD8^+^ immune response against specific tumor-associated antigens (TAA) or tumor-specific antigens (TSA) which highly expressed in cancer cells and not in normal tissues (82). Compared with lentivirus-transduced DC vaccines, peptide vaccines are simple manufacturing and low cost with excellent safety profile. However, the application of single peptide vaccine in clinic seems disappointing. With a better understanding of tumors, it is gradually recognized that tumors are not composed of a single group of cancer cells but have a complex microenvironment including normal tissues and cells as well as immune regulatory network, besides, the immunoevasion of tumors which induces loss of TAA or TSA might render a single peptide vaccine ineffective (83). Moreover, even with the conjunction of adjuvants, the peptide vaccine induced T cell response is far from the antitumor effect with short duration.

## Cancer nucleic acid vaccine

Current cancer nucleic acid vaccines are mainly DNA vaccines and mRNA vaccines. mRNA-based vaccines are well tolerated, easily degradable, and have the potential to induce both humoral and cell-mediated immunity. mRNA-based vaccines are not incorporated into the host genome thereby avoiding carcinogenicity. Dendritic cells (DC) are the target of choice for mRNA vaccine strategies because they link intrinsic and adaptive immune responses and are major regulators of cytotoxic and humoral adaptive responses (84). Synthesized mRNA encoding TAA or TSA is delivered *via* autologous engineered DC with mRNA *in vitro* or *via* formulated or non-formulated (naked) mRNA injections. After vaccination and cellular uptake by APC, mRNA is transported to the cytoplasm for antigen processing and into the presentation of MHC class I and MHC class II to activate CD8^+^ and CD4^+^ T cells. Non-formulated (naked) mRNA vaccines contain only mRNA dissolved in buffer, could be injected administered either intradermally or intranodally. Intranodally administration of non-formulated mRNA enables antigen delivery to APC at the actual site of T-cell activation, thereby avoiding APC migration. A phase I study used an individualized tumor mutation signature with ten selected neoepitopes as target antigen to design mRNA vaccine injected to inguinal lymph nodes under ultrasound control in melanoma patients, all patients developed T-cell responses against numerous vaccine-encoded neoepitopes (85). Non-formulated mRNA vaccines are rarely used in clinic because they are highly susceptible to degradation by RNases in the environment. Therefore, many nanocarrier systems have been developed for application in the production of formulated mRNA vaccines such as protamine-formulated mRNA-based cancer vaccines, mRNA-based lipoplex vaccines, mRNA-based lipid nanoparticle vaccines (86).

Given the ubiquity of RNase and the structural differences between DNA and mRNA, DNA vaccines have longer half-lives than mRNA vaccines. Similarly, DNA vaccines are more heat-stable, allowing for better subcellular sorting and transportation. Because plasmid DNA is more stable than mRNA, there was a large amount of research in the early years of DNA vaccine discovery. In addition, DNA vaccines can be delivered by intratumoral electroporation directly *in situ* in the tumor, thereby driving sufficient antigen processing and presentation (87).

Current strategies for tumor vaccine development focus on the on the following aspects, first, the screening appropriate and specific tumor antigens and effective adjuvants, which may require transcriptome sequencing and proteomics data. Since some tumors are very low in antigenicity, sufficient sequencing depth is required to screen for tumor mutation sites such as single-nucleotide variants (SNVs) and nucleotide insertions or deletions (indels) which result in single amino acid substitutions or aberrant protein expression. Besides, how to product large-scale of nano-vaccine with less economic cost and procedures remains to be resolved. Despite great success made in the animal models, the transformation from animal research to clinical trials still faces many obstacles because of large individual heterogeneity of different patients, as a result, optimal administration of tumor vaccine needs to be explored. Therapeutic vaccines are usually inoculated intravenously while preventive vaccines are usually given subcutaneously and intramuscularly to induce a strong immune response. To achieve a better therapeutic effect, the accessibility of tumor site is also taken into consideration in the selection of the vaccine delivery methods.

## Adoptive cell transfer

ACT isolates natural host T cells that exhibit anti-tumor responsiveness and reinfuses T lymphocytes into patients with the purpose of stimulation and expansion of antigen-specific T cell immunity. Different from one of the significant limitations associated with tumor-vaccine based strategy, ACT doesn’t require *de novo* activate tumor antigen-specific T cell response.

Initial approaches to apply ACT involved leukapheresis of PBMCs from patients followed by bulk ex vivo expansion and reinfusion. CTL and NK cells are majorly exploited as tool cells for the ACT, while DC cells and/or immune effector cells, or combination of both also could be used in ACT. Generally, CTL and NK cells are reinfused along with exogenous IL-2 and DCs are often used as vaccine carriers or APCs to prime naive T cells to be mature effect T cells *in vitro* or *in vivo*. Indeed, ACT does not specifically enrich for antigen-specific T cells, but rather generates a population of non-specific activated T cells which have lowered triggering thresholds. However, comparative analyses revealed that TCR that recognize self-tumor antigens have substantially lower affinities (approximately 1.5 logs) for cognate pMHC complexes compared to their virus-specific counterparts (88), indicating that adopting transfer of autologous T cells may not be sufficient to induce tumor cell death. Therefore, gene transfer-based strategies have been developed to overcome the effects of immune tolerance on tumor-specific T cells. These approaches use genetically engineered host cells with antigen-specific T cell receptor α and β chains (αβTCR) or chimeric antigen receptors (CARs) composed of antigen recognition and binding domains fused to T cell signaling domains. Using these approaches, ACT has mediated significant regression in a variety of cancer tissues, including melanoma, cervical cancer, lymphoma, leukemia, cholangiocarcinoma, and adult neuroblastoma.

## CAR-T cell

In 2017 FDA approved CAR-T cell therapy for the treatment of patients with relapsed or refractory B-acute lymphoblastic leukemia. CAR-T cell therapy involves genetically engineered T cells expressing antigen-specific, non-MHC restricted receptors that can target and attack specific pathological cells and exert therapeutic effects on patients. The structure of CAR is constantly being updated and has now evolved to the fifth generation. The first-generation CAR contains only an extracellular domain which specifically recognizes antigen and an intracellular CD3ζ signaling domain, and their anti-tumor effects are very limited due to the lack of co-stimulatory signals. Second-generation CAR has added an intracellular motif consisting of a co-stimulatory receptor signaling domain to their structure. Even in the absence of exogenous co-stimulatory molecules, second-generation CAR-T cells can continue to proliferate and release cytokines to exert anti-tumor effects. The third-generation CAR contains two co-stimulatory molecules designed to further enhance the killing ability of CAR-T cells. The fourth generation CAR inserts additional molecular components to express functional transgenic proteins, such as interleukin genes or suicide genes, enhancing the killing ability and safety of CAR-T cells. Currently, fifth-generation CAR uses an adapter-specific recognition domain to replace the tumor-specific scFv extracellular structural domain used in previous generations of CAR-T cells, which binds to an adapter molecule that targets a tumor-specific target, such as split, universal, and programmable (SUPRA) CAR system (89) and biotin-binding immune receptor (BBIR) CAR (90).

So far, CAR-T cell immunotherapy has made tremendous progress for hematological malignancies such as acute lymphoblastic leukemia (ALL), diffuse large B-cell lymphoma (DLBCL) and myeloma. The FDA has approved five CAR-T cells, all five targeting B-cell surface markers, four targeting CD19, and one targeting B-cell maturation antigen (BCMA). However, unlike exciting results achieved by CAR-T in hematologic malignancies, the efficacy of CAR-T in solid tumors has been unsatisfactory. Despite the great success of CAR-T targeting CD19 in B-lymphocytic malignancies, there is a lack of antigens targeted on solid tumors. Therefore, target selection is one of the determinants to CAR-T immunotherapy efficacy. For instance, overexpressed proteins on the surface of cancer cells, like mesothelin (MSLN) and epithelial cell adhesion molecule (EpCAM) are highly expressed in a variety of cancers like breast cancers, prostate cancers, and gastric carcinoma, suggesting that they might be good candidate as targets of designed CAR-T cell. Nevertheless, though it is theoretically possible to produce CAR-T cells by gene engineering using mRNA electroporation, typically, transfected mRNA transiently express CAR molecules and produce cytotoxicity up to a week (91), resulting the short lifespan of CAR-T *in vivo*. Besides, some intrinsic characteristics of tumors, such as aberrant vasculature, dense extracellular matrix (ECM) include cancer-associated fibroblast (CAF) and abnormally expressed adhesion molecules, combine to result in inadequate trafficking and infiltration of CAR-T within the tumor. Although some stroma-targeting agents have been considered (92), however, these targets may exist in their host as well. One of CAR-T targeting to CAF was proved to recognize both mouse and human multipotent bone marrow stromal cells (BMSCs) with lethal bone toxicity and cachexia (93). Furthermore, immunosuppressive factors in the TME also hinder the effects of CAR-T cells, like tumor-associated macrophages (TAM), regulatory T (Treg) cells, myeloid-derived suppressor cells (MDSCs) and tumor-associated fibroblasts (TAFs) could directly inhibit CAR-T cells, and many cytokines like transforming growth factor beta (TGF-β), IL-4, IL-10 could promote infiltration of suppressive immune cells, thereby indirectly inhibiting CAR-T. Moreover, when CAR-T cells are fully activated, multiple cytokines would be released including IL-1, IL-2, IL-4, IL-6, IL-8, IL-10, and tumor necrosis factor (TNF) α, the induced cytokine release syndrome (CRS) is severe and even lethal (94). Immune effector cell-associated neurotoxicity syndrome (ICANS) is also observed in some patients under CAR-T therapy (95). Although CAR-T cell activity could be supplemented with inflammatory cytokines such as high dose IL-2, however, systemic IL-2 treatment induces severe capillary leak syndrome and eventually end-organ dysfunction. Allen, Greg M et al. engineered therapeutic T cells bearing synthetic cytokine circuits in which a tumor-specific synthetic Notch (synNotch) receptor drives IL-2 production. In the immune-excluded tumor models like pancreatic cancer and melanoma, engineered synNotch→IL-2 induction circuits induced potent infiltration of chimeric antigen receptor (CAR) or TCR T cells into TME, which provides a possibility of treating solid tumors with CAR-T (96).

## CAR-NK cell

Natural killer (NK) cells are specialized innate immune cells and manifest rapid and potent cytotoxicity for cancer immunotherapy without previous sensitization. NK cells are key mediators of antibody-dependent cell-mediated cytotoxicity (ADCC) recognizing the IgG Fc fraction bound to tumor cells and kill cancer cells by expressing CD16. To avoid graft-versus-host disease (GVHD), CAR-T therapy requires the use of autologous T cells while CAR-NK could be manufactured with “off-the-shelf” cells. Besides, NK cells have a different cytokines profile with T cells which release inflammatory cytokine, leading to CRS, GVHD and neurotoxicity. In a phase I/II trial, the HLA-mismatched anti-CD19 CAR-NK cells derived from cord blood were administered to 11 patients with relapsed or refractory CD19-positive cancers (non-Hodgkin’s lymphoma or chronic lymphocytic leukemia), of the 11 patients treated, 8 (73%) responded. The injected CAR-NK cells expand and persist at low levels for at least 12 months. Besides, the dose of CAR-NK cells was not associated with CRS, GVHD, neurotoxicity and release of cytokines (97). The design of CAR was like CAR-T, consisting of an anti-CD19 scFv extracellular domain, a CD28 transmembrane domain, and a CD28.CD3ζ signaling intracellular domain, in combination with IL-15 gene and the inducible caspase-9 suicide gene. This genetically modified CAR-NK was transduced with a retroviral vector (inducible caspase9/anti-CD19 CAR/IL-15), for the purpose of production of IL-15 to support CAR-NK proliferation and survival, and expression of caspase-9 that can be pharmacologically activated to eliminate transduced cells (98). Human peripheral blood memory-like (ML) NK cells which are modified to express anti-CD19 CAR (19-CAR-ML) exhibited enhanced NK cell functional responses (cytotoxicity, degranulation, and cytokine production) which were CAR-antigen specific. Besides, in NSG (NOD-scid IL-2Rγ^null^) mice inoculated IV with Raji cells, 19-CAR-ML NK cells were able to effectively expand and persist, control tumor growth, and prolong survival of tumor-bearing mice (99).

Although the advantages of CAR-NK cell therapy over CAR-T cell therapy are clear, there are significant limitations. Almost all the hurdles associated with CAR-T therapy also apply to CAR-NK cells, from target selection, CAR design, manufacturing to post-infusion challenges such as difficulties in migration to tumor sites, and immunosuppressive TME. In addition, NK cells have a short half-life, which is a double-edged sword, which means that CAR-NK is advantageous in the event of severe toxicity, but also poses a challenge in that repeated dosing may be required to achieve a durable response. The reprogramming of CAR-NK cells with memory cell properties and long-term survival *in vivo* for continuous immune surveillance and prevention of cancer recurrence is currently an area of active exploration.

## CAR-macrophage

Due to barriers to CAR-T and CAR-NK cell therapies, CAR-macrophage (CAR-M) research has emerged as an alternative therapy. Unlike CAR-T, CAR-M have high trafficking and infiltration within solid tumors while T cells are physically excluded or inactivated (**Table 2**). Tumor-associated macrophages (TAMs) derive from circulating monocytic precursors, infiltrating macrophages in tumor tissue are driven by tumor-derived cytokines (e.g. IL-10, TGF-β) and T cell-derived cytokines (e.g. IL-4, IL-13) and acquire a polarized M2 phenotype (100). TAMs assist in tumor growth, cancer immunosuppression, and angiogenesis. High infiltration rate of TAMs usually associated with poor prognosis in solid tumor, and TAMs interact with almost all cellular components of the TME (including cancer cells, immune cells and other resident non-immune cells), therefore, development of CAR-M has great promise. Most CAR-M only engulf fragments of target cells, a phenomenon resembling trogocytosis while the whole cell engulfment is infrequent. There have been studies confirming that blockade of the ‘don’t-eat-me’ signal CD47 (101) or CAR containing designed intercellular domain, like derived from FcRγ, multiple EGF-like-domains protein 10 (Megf10), and the CD19 cytoplasmic domain that recruits the p85 subunit of phosphoinositide-3 kinase (PI3K) (102) could enhance macrophage phagocytic capacity.

**Table 2:** Comparations of CAR- T cells, CAR-NK cells and CAR-macrophages.

	CAR-T CELLS	CAR-NK CELLS	CAR-MACROPHAGES
Structure of CAR	An adapter-specific recognition extracellular domain, a transmembrane domain and an intracellular domain CD3ζ with an intracellular co-stimulatory motif	A scFv extracellular domain, a CD28 transmembrane domain and an intracellular domain CD3ζ	A scFv extracellular domain, a CD8 transmembrane domain and an intracellular domain like FcRγ, Megf10 and CD19 cytoplasmic domain
Origin source	Autologous T cells or MHC-matched allogeneic	Autologous NK cells, non-MHC-matched allogeneic or NK cell lines (off-the-shelf)	Autologous macrophages. Or iPSCs and cell lines (Theoretically off-the-shelf, but only in preclinical studies, no clinical data available)
Anti-tumor mechanisms	CAR-dependent CTL effect	CAR-dependent ADCC effect	CAR-dependent phagocytosis of macrophages; differentiation of macrophages into a pro-inflammatory M1-like phenotype.
CRS and ICANS	Common and severe	Less common but also severe	No clinical data available
Reference	(90, 94)	(97, 98)	(103, 104)

Like CAR-T, the core components of CAR-M contain an extracellular structural domain that provides specific recognition through single-chain variable regions (scFv), a hinge structural domain, a transmembrane structural domain (mainly CD8), and an intracellular structural domain that provides dedicated downstream signaling which is usually associated with activation and enhancement of the phagocytic effect. Current studies on extracellular signaling domains have identified several common tumor targets, such as CD19 and HER2. The design of intracellular signaling domains of CAR-M are quite diverse, and alternative domains have been explored by several groups. By screening a panel of engulfment receptor intracellular domains, Morrissey, M. A., et al. engineered a kind of CAR for phagocytosis containing Megf10, FcRγ, adhesion G protein-coupled receptor B1 (Bai1) and tyrosine-protein kinase Mer (MerTK) which direct macrophages to engulf specific antigen (103). To breakdown the ‘physical barrier’ of the tumor ECM, Zhang, W., et al. designed CAR-M contains CD147 which is essential for ECM remodeling by expressing MMPs (104).

TAM is known to be the polarized anti-inflammatory M2 phenotype which is considered to promote tissue remodeling and tumor growth, consequently leads to its immunosuppressive function. Zhang, L., et al. used non-integrating episomal vectors encoding reprograming factors to induce pluripotent stem cells and introduced CAR into single induced pluripotent stem cells (iPSC) clones *via* lentiviral transduction to obtain CAR-iPSC and then established a protocol for myeloid/macrophage differentiation to induce differentiation of CAR-iPSCs to myeloid cells to obtain CAR-expressing iPSC-induced macrophage (CAR-iMac). In this research, CAR-iMac also possessed M2 phenotype, however, in NSG mice, when CAR-iMac cells were treated with IFN-γ to polarize toward pro-inflammatory M1 phenotype before injection, CAR-iMac-treated mice showed reduced tumor burden compared to controls (105). Klichinsky, M., et al. found that CAR-M induced pro-inflammatory pathways such as IFN signaling, TH1 pathway and iNOS signaling in M2 macrophages, expressing qe1pro-inflammatory cytokines and chemokines that phenotypically convert M2 macrophages to M1, while inducing activation and maturation markers in immature DCs to upregulate antigen presentation mechanisms and recruit both resting and activated T cells to resist immunosuppressive cytokines (106).

Despite the significant advantages of CAR-M over CAR-T, its application in the clinic remains limited. First, macrophages which are highly differentiated cells, do not have a proliferative potential either *in vitro* or after injection *in vivo*. Besides, the complex TME should also be considered when applying CAR-M therapy. Due to the heterogeneity of tumor cells, the selection of targets of CAR-M would be also difficult.

In general, CAR-T therapy has made tremendous progress in hematological malignancies while CAR-T therapy in solid tumors has unsatisfactory effect due to lack of cancer-specific antigen, low cell trafficking of CAR-T in TME and migration into tumor sites, immunosuppressive TME among others. Besides, on account of the strict MHC restriction and strong ability to release cytokines, CAR-T therapy probably induces severe GVHD, CRS and ICANS. However, different cytokines profile and limited life span make CAR-NK a diminished risk for inducing GVHD, CRS and ICANS, and non-MHC restriction allows generation of off-the-shelf allogeneic CAR-NK cells using NK cell lines. Macrophages are the major infiltrated cells and mainly immune regulators in TME. Even TAM are immunosuppressive M2 phenotype, they also possess strong phagocytic activity, indicting a strong potential for engineered CAR-macrophage. There has been CAR-macrophages explored as an alternative approach for the ACT. Future CAR macrophage therapy still needs to overcome some other obstacles encountered with CAR T therapy. Since TAM is also significant immune regulator, one major research direction is to develop CAR-macrophage not only as a phagocytic executor but an antigen presenter and immune stimulator to promote anticancer immunity. But at present only preclinical data is available.

## Oncolytic virus therapy

**Figure 4:**
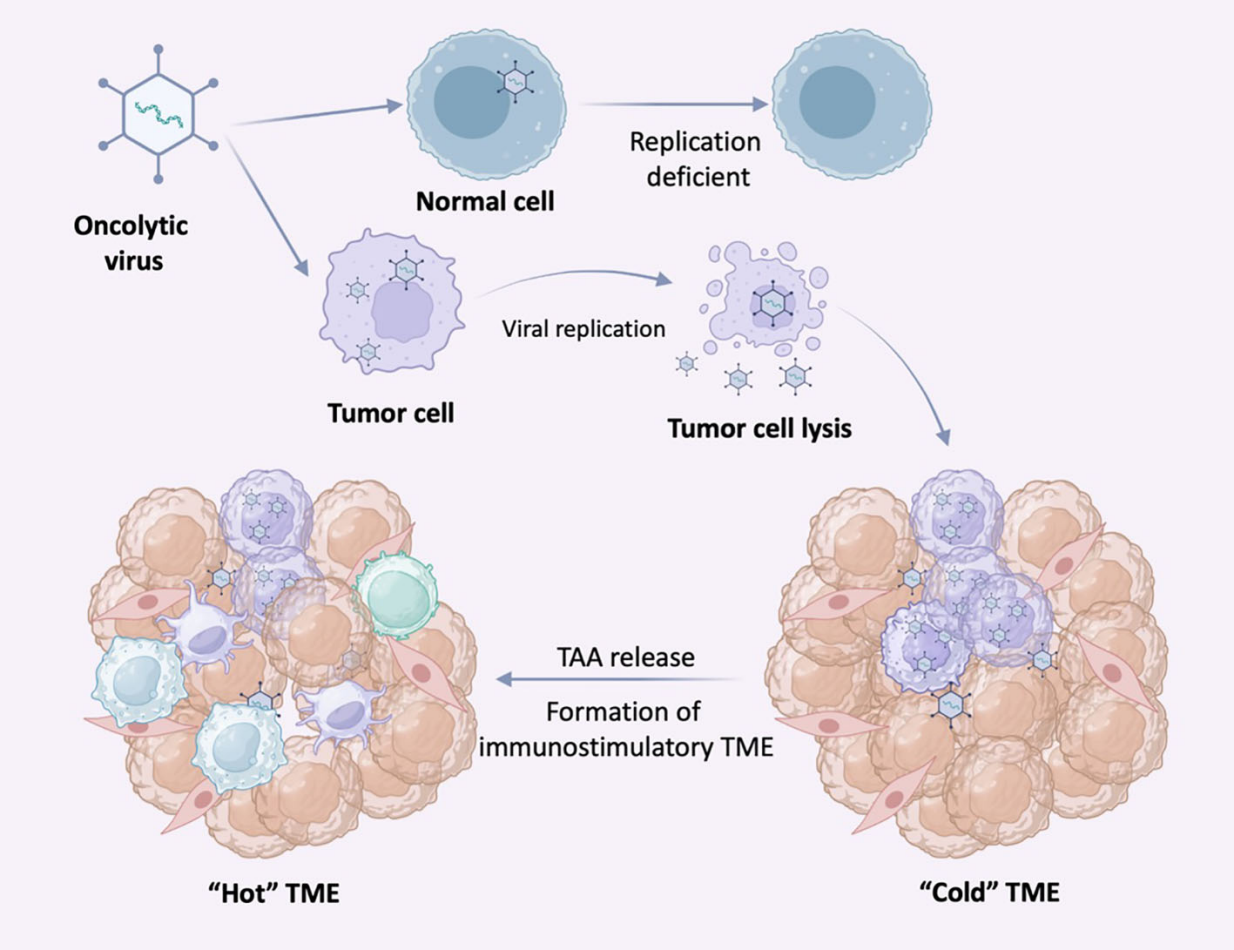
Selective replication of oncolytic virus (OVs) in tumor cells. OVs can specifically infect tumor cells and replicate in tumor cells until the tumor cells lyse and release nascent virus to infect neighboring tumor cells. In normal cells, OVs replicate at low or no levels due to antiviral signaling and other mechanisms. Following infection with OV, tumor cells release antiviral cytokines to initiate an antiviral response.

Oncolytic viruses (OVs) are replication-specific viruses that directly infect and lyse tumor cells in situ. OVs can enter both normal and cancer cells, but the inherent abnormalities in the cancer cell provide a selective advantage for viral replication, allowing replication within tumor cells and direct lysis of tumor cells, promoting tumor antigen presentation to induce systemic anti-tumor immunity. Since virus recognition and clearance mechanisms such as IFN release, Toll-like receptors (TLRs) signaling, and PKR-related pathways exist in normal cells, these pathways may be abnormal in tumor cells. Following infection with OV, tumor cells release antiviral cytokines (e.g. IFN) to initiate an antiviral response. After tumor cell lysis, viral progeny, tumor-associated antigens (TAA) including neoantigens, are released. Antiviral cytokines promote maturation of antigen-presenting cells (APCs), and viral progeny infect more tumor cells. TAA and neoantigens are taken up by APC and activate antigen/virus-specific CD8^+^ T cell responses, thus creating an immune-stimulating environment (**Figure 4**). Both the changes in type of cell death and danger signals released by virus-infected cells can largely aid in the induction of host immune responses. For example, necrosis or pyroptosis is a more immunogenic form of cell death than apoptosis (107). Up to date, four OVs have been approved to treat various cancers: Rigvir, T-VEC (IMLYGIC), ONYX-015 (dl1520), and H101.

Rigvir is an unmodified virus belonging to the *Picornaviridae* family, Enterovirus genus, ECHO group, type 7, as the first OV, it was approved in Latvia in 2004 for local treatment of skin and subcutaneous metastases of melanoma for prevention of relapse and metastasis after radical surgery (108). Clinical studies also intend to identify markers and expand the range of indications of Rigvir since it has less severe adverse events. One of the latest Trends in the field of oncology treatment is combination therapy, thus adding a potential therapeutic mode for the application of Rigvir, such as testing the effects of combining anti-PD-1 antibodies with Rigvir (109). T-VEC is a genetically modified type 1 herpes simplex virus (HSV-1) which is the first approved oncolytic virus for the treatment of advanced melanoma by the US FDA (110) and remains the most widely approved therapy up to date. Deletion of the herpes neurovirulence virus gene and deletion of the viral ICP47 gene resulted in reduced virulence and increased immunogenicity of T-VEC, while T-VEC can encode GM-CSF to stimulate the host immune system (110). In a phase III clinical trial, the T-VEC arm had significantly higher durable response rate (16.3%; 95% CI, 12.1% to 20.5% versus 2.1%; 95% CI, 0% to 4.5%) and OS (26.4%; 95% CI, 21.4% to 31.5% versus 5.7%; 95% CI, 1.9% to 9.5%) compared to GM-CSF arm (111).

Restoration of wild-type p53 function in tumors with an exogenous vector (in most cases an adenoviral vector) is a kind of gene replacement therapies that has been shown to inhibit tumor growth. An advantage of the adenovirus delivery system is that it does not result in integration of the vector DNA into the host cells. ONYX-015 is an adenovirus lacking a 55 kDa protein from the E1B region and was first designed to activate p53 function as E1B could bind and inactive p53 (112). ONYX-015 is currently the most prominent and clinically evaluated p53-expressing conditionally replicating adenovirus vectors (CRAdp53) which can only proliferate effectively in p53 mutant tumor cells but not p53 wild-type cells. Although there’s some evidence of antitumor activity for ONYX-015, the clinical effect of it has varied greatly, and there are still many patients responding poorly (113). H101 is also an E1b55K-deleted adenovirus with an additional deletion of 78.3–85.8 μm gene segment in the E3 region. Chinese government regulators (SFDA) approved H101 especially for advanced nasopharyngeal carcinoma in combination with cisplatin and 5-FU chemotherapy in 2005 (114).

Given the tolerable safety profile of OVs, they are interesting agents to use in combination approaches. With the ability of viral infection to induce IFN secretion, promoting cell necrosis and the release of damage-associated molecular patterns (DAMPs), OVs are attractive as combination agents with other forms of tumor immunotherapy. As mentioned earlier, tumors can be classified into “cold” and “hot” tumors according to the immune infiltration within the tumor, in which “hot” tumors are much more responsive to ICIs. Given the characteristic expression profile of IFNs in tumors might induced by OVs, it is suggested that OVs may serve as an inducer of tumors to become “hot”. OV infection enhances the infiltration and activity of both innate and adaptive immune cells within the TME (**Figure 4**). To date, the most studies are still focused on the combination of OVs and ICIs.

As other immunotherapies, OVs also have some limitations. First, there is a potential for host to produce neutralizing antibodies, besides, in the hypoxia tumor core, tumor cells would form nearby necrosis or calcification in response to hypoxia or acidosis, which might limit the efficacy of OVs. Differently from most other anticancer therapeutics, OVs are live replicating virus which include unique challenge. Furthermore, although OVs could rapidly kill and lyse tumor cells *in situ* and expose internal antigens, too fast lysis of cells may be detrimental to viral expansion, as cells are lysed before an optimal quantity of daughter viruses being replicated. Besides, OVs are administered to patients intratumorally, rendering many of the traditional methods for establishing clinical trial eligibility endpoints, pharmacokinetics, and dosing inappropriate for the evaluation of OVs.

## Discussions and perspectives

With the emerging field of synthetic immunology and the progressive understanding of TME, the application of immunotherapy in the field of tumor treatment has been flourishing and gradually becoming a new approach to transform tumor treatment. Several immune checkpoint inhibitors have been approved to apply into clinical treatment and have satisfactory outcome, but some tumor patients do not respond to ICIs because of low expression of immune checkpoints and tumor cell intrinsic resistance. Tumor vaccines targeting specific tumor-associated antigens (TAA) or tumor-specific antigens (TSA) have made some progress in preventing or treating kind of tumors like Gardasil for uterine cancer and sipuleucel-T for advanced prostate cancer. However, most tumors have low antigenicity and no suitable TSA or TAA could be selected to design vaccines. Besides, manufacturing nanocarriers of tumor vaccine needs complex procedures and expensive materials which make this approach time-consuming and economy-costing. What’s more, due to the different immune systems among different individuals, it’s difficult to transform animal vaccine research to human trials. Adoptive cell transfer using genetically modified T cells recognizing specific pMHC complexes has significantly improved the ability of T cells killing malignant hematopoietic tumor cells, but induced graft-versus-host disease (GVHD) and cytokine release syndrome (CRS) is severe and sometimes even lethal. Under these circumstances, MHC-free restricted NK cells and macrophages were also transgenically modified. Under these circumstances, non-MHC restricted NK cells and macrophages were also genetically modified, demonstrating lower side effects, but still lacking sufficient available clinical data. Oncolytic virus immunotherapy utilizes native or genetically modified viruses that has selective replication advantages within tumor cells. The ability of viruses to direct kill cancer cells has been recognized for nearly a century, but only over the past decade have clinical trials authenticated a therapeutic benefit of oncolytic virus in patients with cancer, which made oncolytic virus therapy a new class of immunotherapy. Melanoma patients treated with T-VEC showed significantly improved durable responses (111), which has revolutionized the field of OVs anti-cancer therapy.

Although the efficacy of ICIs in many cancers is encouraging, there are still some difficulties. First, ICIs are effective only in highly immune infiltrated cancers, but not in “cold” tumors with little immune cells infiltration, such as pancreatic ductal adenocarcinoma (PDAC). Second, due to cancer heterogeneity and individual patient differences, the response to ICIs varies among patients with the same cancer, and some patients still show no response. Therefore, for “cold” tumors refractory to ICIs, cancer vaccines or oncolytic virus in combination with ICIs could be considered in clinical trials, aiming to improve TSA or TAA exposure and immune cell infiltration in “cold” tumors and further enhance the immune system’s attack on the tumor system. In view of heterogeneity of tumor cells and complexity of TME, individualized or personalized therapy might be alternative approach. For some patients who do not respond to ICIs, further clinical identification of reliable biomarkers is needed to help determine which patients are suitable for treatment with ICIs. Finally, although some patients with high expression of immune checkpoints, such as PD-L1, did not have an elevated ORR after ICIs (115), suggesting that there may be some protein modifications that make this group of patients unresponsive to ICIs but included in the population suitable for ICIs.

Cancer vaccines have shown great promise for cancer immunotherapy in preclinical studies because of their ability to provide precision treatment for individuals. However, some limitations need to be urgently considered. In terms of mechanistic studies, vaccine inventions aim at activating immune response mechanisms, so the screening of new antigens is crucial and cancer vaccines that mimic the characteristics of autologous pathogens are needed to target tumor cells for precise and individualized treatment. With the booming development of bioinformatics, genomic informatics, transcriptome sequencing technologies and proteomics can be used to find suitable antigens. Also, suitable vectors can be developed for cancer vaccine delivery. Regarding clinical translation, there is a lack of suitable animal models to study the role of cancer vaccines in humans, particularly the balance between immune defense and systemic hyperactivation after vaccination.

Despite the great progress of CAR-T therapy in hematopoietic malignancies, the efficacy of CAR - T for solid tumors is poor: the lack of tumor-associated antigens, immunosuppressive TME and other problems lead to solid tumors not responding to CAR-T therapy. The development of CAR-macrophages is currently underway to improve suppressive TME, and the development of novel delivery modalities to improve the efficiency of CAR-T, CAR-NK, and CAR-macrophages transport and migration to target cells *in vivo* is of high clinical relevance.

Discovery of oncolytic virus anti-tumor mechanism provides new viable options for tumor therapy. The discovery of the anti-tumor mechanism of lysovirus provides a new viable option for tumor treatment. However, the anti-tumor mechanisms of OVs have not been fully elucidated, and secondly, the short half-life of OVs and the limited viral replication and dissemination in TME limit the efficacy of OVs. Considering the low efficacy of OVs monotherapy and the safety of systemic therapy, OVs are usually used in combination with ICIs, chemotherapy drugs or other therapeutic regimens.

## Author contributions

RR wrote the manuscript. LZ and SH provided the financial support and reviewed the manuscript. All authors contributed to the article and approved the submitted version.

## References

[1] Dunn GP Bruce AT Ikeda H Old LJ Schreiber RD. Cancer immunoediting: from immunosurveillance to tumor escape. Nat Immunol (2002) 3:991–8. doi: 10.1038/ni1102-991 12407406

[2] Hanahan D Weinberg RA. Hallmarks of cancer: the next generation. Cell (2011) 144:646–74. doi: 10.1016/j.cell.2011.02.013 21376230

[3] Vinay DS Ryan EP Pawelec G Talib WH Stagg J Elkord E. Immune evasion in cancer: Mechanistic basis and therapeutic strategies. Semin Cancer Biol (2015) 35(Suppl):S185–s198. doi: 10.1016/j.semcancer.2015.03.004 25818339

[4] Loi S Michiels S Adams S Loibl S Budczies J Denkert C. The journey of tumor-infiltrating lymphocytes as a biomarker in breast cancer: clinical utility in an era of checkpoint inhibition. Ann Oncol (2021) 32:1236–44. doi: 10.1016/j.annonc.2021.07.007 34311075

[5] Duan Q Zhang H Zheng J Zhang L. Turning Cold into Hot: Firing up the Tumor Microenvironment. Trends Cancer (2020) 6:605–18. doi: 10.1016/j.trecan.2020.02.022 32610070

[6] Tawbi HA SChadendorf D Lipson EJ Ascierto PA Matamala L Castillo Gutiérrez E. Relatlimab and nivolumab versus nivolumab in untreated advanced melanoma. N Engl J Med (2022) 386:24–34. doi: 10.1056/NEJMoa2109970 34986285 PMC9844513

[7] Niu J Maurice-Dror C Lee DH Kim DW Nagrial A Voskoboynik M. First-in-human phase 1 study of the anti-TIGIT antibody vibostolimab as monotherapy or with pembrolizumab for advanced solid tumors, including non-small-cell lung cancer(☆). Ann Oncol (2022) 33:169–80. doi: 10.1016/j.annonc.2021.11.002 34800678

[8] Kaptein P Jacoberger-Foissac C Dimitriadis P Voabil P de Bruijn M Brokamp S. Addition of interleukin-2 overcomes resistance to neoadjuvant CTLA4 and PD1 blockade in ex vivo patient tumors. Sci Transl Med (2022) 14:eabj9779. doi: 10.1126/scitranslmed.abj9779 35476594

[9] Wherry EJ. T cell exhaustion. Nat Immunol (2011) 12:492–9. doi: 10.1038/ni.2035 21739672

[10] Larkin J Minor D D'Angelo S Neyns B Smylie M Miller WH Jr.. Overall survival in patients with advanced melanoma who received nivolumab versus investigator's choice chemotherapy in checkMate 037: A randomized, controlled, open-label phase III trial. J Clin Oncol (2018) 36:383–90. doi: 10.1200/jco.2016.71.8023 PMC6804912 28671856

[11] Hodi FS O'Day SJ McDermott DF Weber RW Sosman JA Haanen JB. Improved survival with ipilimumab in patients with metastatic melanoma. N Engl J Med (2010) 363:711–23. doi: 10.1056/NEJMoa1003466 PMC3549297 20525992

[12] Weber JS D'Angelo SP Minor D Hodi FS Gutzmer R Neyns B. Nivolumab versus chemotherapy in patients with advanced melanoma who progressed after anti-CTLA-4 treatment (CheckMate 037): a randomised, controlled, open-label, phase 3 trial. Lancet Oncol (2015) 16:375–84. doi: 10.1016/s1470-2045(15)70076-8 25795410

[13] Rizvi NA Mazières J Planchard D Stinchcombe TE Dy GK Antonia SJ. Activity and safety of nivolumab, an anti-PD-1 immune checkpoint inhibitor, for patients with advanced, refractory squamous non-small-cell lung cancer (CheckMate 063): a phase 2, single-arm trial. Lancet Oncol (2015) 16:257–65. doi: 10.1016/s1470-2045(15)70054-9 PMC5726228 25704439

[14] Motzer RJ Escudier B McDermott DF George S Hammers HJ Srinivas S. Nivolumab versus everolimus in advanced renal-cell carcinoma. N Engl J Med (2015) 373:1803–13. doi: 10.1056/NEJMoa1510665 PMC5719487 26406148

[15] Armand P Engert A Younes A Fanale M Santoro A Zinzani PL. Nivolumab for relapsed/refractory classic hodgkin lymphoma after failure of autologous hematopoietic cell transplantation: extended follow-up of the multicohort single-arm phase II checkMate 205 trial. J Clin Oncol (2018) 36:1428–39. doi: 10.1200/jco.2017.76.0793 PMC6075855 29584546

[16] Robert C Schachter J Long GV Arance A Grob JJ Mortier L. Pembrolizumab versus ipilimumab in advanced melanoma. N Engl J Med (2015) 372:2521–32. doi: 10.1056/NEJMoa1503093 25891173

[17] Paz-Ares L Vicente D Tafreshi A Robinson A Soto Parra H Mazières J. A randomized, placebo-controlled trial of pembrolizumab plus chemotherapy in patients with metastatic squamous NSCLC: protocol-specified final analysis of KEYNOTE-407. J Thorac Oncol (2020) 15:1657–69. doi: 10.1016/j.jtho.2020.06.015 32599071

[18] Rini BI Plimack ER Stus V Gafanov R Hawkins R Nosov D. Pembrolizumab plus Axitinib versus Sunitinib for Advanced Renal-Cell Carcinoma. N Engl J Med (2019) 380:1116–27. doi: 10.1056/NEJMoa1816714 30779529

[19] Chen R Zinzani PL Fanale MA Armand P Johnson NA Brice P. Phase II study of the efficacy and safety of pembrolizumab for relapsed/refractory classic hodgkin lymphoma. J Clin Oncol (2017) 35:2125–32. doi: 10.1200/jco.2016.72.1316 PMC5791843 28441111

[20] Socinski MA Jotte RM Cappuzzo F Orlandi F Stroyakovskiy D Nogami N. Atezolizumab for first-line treatment of metastatic nonsquamous NSCLC. N Engl J Med (2018) 378:2288–301. doi: 10.1056/NEJMoa1716948 29863955

[21] Balar AV Galsky MD Rosenberg JE Powles T Petrylak DP Bellmunt J. Atezolizumab as first-line treatment in cisplatin-ineligible patients with locally advanced and metastatic urothelial carcinoma: a single-arm, multicentre, phase 2 trial. Lancet (2017) 389:67–76. doi: 10.1016/s0140-6736(16)32455-2 27939400 PMC5568632

[22] Schmid P Adams S Rugo HS Schneeweiss A Barrios CH Iwata H. Atezolizumab and nab-paclitaxel in advanced triple-negative breast cancer. N Engl J Med (2018) 379:2108–21. doi: 10.1056/NEJMoa1809615 30345906

[23] Cho BC Abreu DR Hussein M Cobo M Patel AJ Secen N. Tiragolumab plus atezolizumab versus placebo plus atezolizumab as a first-line treatment for PD-L1-selected non-small-cell lung cancer (CITYSCAPE): primary and follow-up analyses of a randomised, double-blind, phase 2 study. Lancet Oncol (2022) 23:781–92. doi: 10.1016/s1470-2045(22)00226-1 35576957

[24] Fraser JD Irving BA Crabtree GR Weiss A. Regulation of interleukin-2 gene enhancer activity by the T cell accessory molecule CD28. Science (1991) 251:313–6. doi: 10.1126/science.1846244 1846244

[25] Rudd CE Taylor A Schneider H. CD28 and CTLA-4 coreceptor expression and signal transduction. Immunol Rev (2009) 229:12–26. doi: 10.1111/j.1600-065X.2009.00770.x 19426212 PMC4186963

[26] Collins AV Brodie DW Gilbert RJ Iaboni A Manso-Sancho R Walse B. The interaction properties of costimulatory molecules revisited. Immunity (2002) 17:201–10. doi: 10.1016/s1074-7613(02)00362-x 12196291

[27] Qureshi OS Kaur S Hou TZ Jeffery LE Poulter NS Briggs Z. Constitutive clathrin-mediated endocytosis of CTLA-4 persists during T cell activation. J Biol Chem (2012) 287:9429–40. doi: 10.1074/jbc.M111.304329 PMC3308817 22262842

[28] Qureshi OS Zheng Y Nakamura K Attridge K Manzotti C Schmidt EM. Trans-endocytosis of CD80 and CD86: a molecular basis for the cell-extrinsic function of CTLA-4. Science (2011) 332:600–3. doi: 10.1126/science.1202947 PMC3198051 21474713

[29] Kooh PJ Fehon RG Muskavitch MA. Implications of dynamic patterns of Delta and Notch expression for cellular interactions during Drosophila development. Development (1993) 117:493–507. doi: 10.1242/dev.117.2.493 8330521

[30] Kennedy A Waters E Rowshanravan B Hinze C Williams C Janman D. Differences in CD80 and CD86 transendocytosis reveal CD86 as a key target for CTLA-4 immune regulation. Nat Immunol (2022) 23:1365–78. doi: 10.1038/s41590-022-01289-w PMC9477731 35999394

[31] Quigley M Pereyra F Nilsson B Porichis F Fonseca C Eichbaum Q. Transcriptional analysis of HIV-specific CD8+ T cells shows that PD-1 inhibits T cell function by upregulating BATF. Nat Med (2010) 16:1147–51. doi: 10.1038/nm.2232 PMC3326577 20890291

[32] Leach DR Krummel MF Allison JP. Enhancement of antitumor immunity by CTLA-4 blockade. Science (1996) 271:1734–6. doi: 10.1126/science.271.5256.1734 8596936

[33] Wolchok JD Neyns B Linette G Negrier S Lutzky J Thomas L. Ipilimumab monotherapy in patients with pretreated advanced melanoma: a randomised, double-blind, multicentre, phase 2, dose-ranging study. Lancet Oncol (2010) 11:155–64. doi: 10.1016/s1470-2045(09)70334-1 20004617

[34] Arce Vargas F Furness AJS Litchfield K Joshi K Rosenthal R Ghorani E. Fc effector function contributes to the activity of human anti-CTLA-4 antibodies. Cancer Cell (2018) 33:649–663.e644. doi: 10.1016/j.ccell.2018.02.010 29576375 PMC5904288

[35] Peggs KS Quezada SA Chambers CA Korman AJ Allison JP. Blockade of CTLA-4 on both effector and regulatory T cell compartments contributes to the antitumor activity of anti-CTLA-4 antibodies. J Exp Med (2009) 206:1717–25. doi: 10.1084/jem.20082492 PMC2722174 19581407

[36] Yofe I Landsberger T Yalin A Solomon I Costoya C Demane DF. Anti-CTLA-4 antibodies drive myeloid activation and reprogram the tumor microenvironment through FcgammaR engagement and type I interferon signaling. Nat Cancer (2022) 3:1336–50. doi: 10.1038/s43018-022-00447-1 36302895

[37] Day D Hansen AR. Immune-related adverse events associated with immune checkpoint inhibitors. BioDrugs (2016) 30:571–84. doi: 10.1007/s40259-016-0204-3 27848165

[38] Dovedi SJ Elder MJ Yang C Sitnikova SI Irving L Hansen A. Design and efficacy of a monovalent bispecific PD-1/CTLA4 antibody that enhances CTLA4 blockade on PD-1(+) activated T cells. Cancer Discovery (2021) 11:1100–17. doi: 10.1158/2159-8290.CD-20-1445 33419761

[39] Sharpe AH Pauken KE. The diverse functions of the PD1 inhibitory pathway. Nat Rev Immunol (2018) 18:153–67. doi: 10.1038/nri.2017.108 28990585

[40] Wherry EJ Ha SJ Kaech SM Haining WN Sarkar S Kalia V. Molecular signature of CD8+ T cell exhaustion during chronic viral infection. Immunity (2007) 27:670–84. doi: 10.1016/j.immuni.2007.09.006 17950003

[41] Kurtulus S Madi A Escobar G Klapholz M Nyman J Christian E. Checkpoint blockade immunotherapy induces dynamic changes in PD-1(-)CD8(+) tumor-infiltrating T cells. Immunity (2019) 50:181–194 e186. doi: 10.1016/j.immuni.2018.11.014 30635236 PMC6336113

[42] Larkin J Chiarion-Sileni V Gonzalez R Grob JJ Cowey CL Lao CD. Combined nivolumab and ipilimumab or monotherapy in untreated melanoma. N Engl J Med (2015) 373:23–34. doi: 10.1056/NEJMoa1504030 26027431 PMC5698905

[43] Borghaei H Paz-Ares L Horn L Spigel DR Steins M Ready NE. Nivolumab versus docetaxel in advanced nonsquamous non-small-cell lung cancer. N Engl J Med (2015) 373:1627–39. doi: 10.1056/NEJMoa1507643 PMC5705936 26412456

[44] Brahmer J Reckamp KL Baas P Crinò L Eberhardt WE Poddubskaya E. Nivolumab versus docetaxel in advanced squamous-cell non-small-cell lung cancer. N Engl J Med (2015) 373:123–35. doi: 10.1056/NEJMoa1504627 PMC4681400 26028407

[45] Ansell SM Lesokhin AM Borrello I Halwani A Scott EC Gutierrez M. PD-1 blockade with nivolumab in relapsed or refractory Hodgkin's lymphoma. N Engl J Med (2015) 372:311–9. doi: 10.1056/NEJMoa1411087 PMC4348009 25482239

[46] Workman CJ Vignali DA. Negative regulation of T cell homeostasis by lymphocyte activation gene-3 (CD223). J Immunol (2005) 174:688–95. doi: 10.4049/jimmunol.174.2.688 15634887

[47] Workman CJ Dugger KJ Vignali DA. Cutting edge: molecular analysis of the negative regulatory function of lymphocyte activation gene-3. J Immunol (2002) 169:5392–5. doi: 10.4049/jimmunol.169.10.5392 12421911

[48] Iouzalen N Andreae S Hannier S Triebel F. LAP, a lymphocyte activation gene-3 (LAG-3)-associated protein that binds to a repeated EP motif in the intracellular region of LAG-3, may participate in the down-regulation of the CD3/TCR activation pathway. Eur J Immunol (2001) 31:2885–91. doi: 10.1002/1521-4141(2001010)31:10<2885::aid-immu2885>3.0.co;2-2 11592063

[49] Triebel F Jitsukawa S Baixeras E ROman-ROman S Genevee C Viegas-Pequignot E. LAG-3, a novel lymphocyte activation gene closely related to CD4. J Exp Med (1990) 171:1393–405. doi: 10.1084/jem.171.5.1393 PMC2187904 1692078

[50] Xu F Liu J Liu D Liu B Wang M Hu Z. LSECtin expressed on melanoma cells promotes tumor progression by inhibiting antitumor T-cell responses. Cancer Res (2014) 74:3418–28. doi: 10.1158/0008-5472.Can-13-2690 24769443

[51] Wang J Sanmamed MF Datar I Su TT Ji L Sun J. Fibrinogen-like protein 1 is a major immune inhibitory ligand of LAG-3. Cell (2019) 176:334–347.e312. doi: 10.1016/j.cell.2018.11.010 30580966 PMC6365968

[52] Maruhashi T Sugiura D Okazaki IM Shimizu K Maeda TK Ikubo J. Binding of LAG-3 to stable peptide-MHC class II limits T cell function and suppresses autoimmunity and anti-cancer immunity. Immunity (2022) 55:912–924 e918. doi: 10.1016/j.immuni.2022.03.013 35413245

[53] Amaria RN Postow M Burton EM Tezlaff MT Ross MI Torres-Cabala C. Neoadjuvant relatlimab and nivolumab in resectable melanoma. Nature (2022) 611:155–60. doi: 10.1038/s41586-022-05368-8 PMC9607737 36289334

[54] Levin SD Taft DW Brandt CS Bucher C Howard ED Chadwick EM. Vstm3 is a member of the CD28 family and an important modulator of T-cell function. Eur J Immunol (2011) 41:902–15. doi: 10.1002/eji.201041136 PMC3733993 21416464

[55] Xu Z Jin B. A novel interface consisting of homologous immunoglobulin superfamily members with multiple functions. Cell Mol Immunol (2010) 7:11–9. doi: 10.1038/cmi.2009.108 PMC4003259 20081873

[56] Stanietsky N Simic H Arapovic J Toporik A Levy O Novik A. The interaction of TIGIT with PVR and PVRL2 inhibits human NK cell cytotoxicity. Proc Natl Acad Sci (2009) 106:17858–63. doi: 10.1073/pnas.0903474106 PMC2764881 19815499

[57] Chiang EY Mellman I. TIGIT-CD226-PVR axis: advancing immune checkpoint blockade for cancer immunotherapy. J Immunother Cancer (2022) 10. doi: 10.1136/jitc-2022-004711 PMC8981293 35379739

[58] Stengel KF Harden-Bowles K Yu X Rouge L Yin J Comps-Agrar L. Structure of TIGIT immunoreceptor bound to poliovirus receptor reveals a cell-cell adhesion and signaling mechanism that requires cis-trans receptor clustering. Proc Natl Acad Sci U S A. (2012) 109:5399–404. doi: 10.1073/pnas.1120606109 PMC3325733 22421438

[59] Dolgin E. Antibody engineers seek optimal drug targeting TIGIT checkpoint. Nat Biotechnol (2020) 38:1007–9. doi: 10.1038/s41587-020-0666-1 32887966

[60] An anti-TIGIT antibody with a PD-1 inhibitor shows promise in solid tumors. Cancer Discovery (2022) 12:14. doi: 10.1158/2159-8290.Cd-rw2021-170 34819317

[61] Oldenborg PA Gresham HD Lindberg FP. CD47-signal regulatory protein alpha (SIRPalpha) regulates Fcgamma and complement receptor-mediated phagocytosis. J Exp Med (2001) 193:855–62. doi: 10.1084/jem.193.7.855 PMC2193364 11283158

[62] Oldenborg PA Zheleznyak A Fang YF Lagenaur CF Gresham HD Lindberg FP. Role of CD47 as a marker of self on red blood cells. Science (2000) 288:2051–4. doi: 10.1126/science.288.5473.2051 10856220

[63] Zhao XW van Beek EM Schornagel K van der Maaden H Van Houdt M Otten MA. CD47-signal regulatory protein-alpha (SIRPalpha) interactions form a barrier for antibody-mediated tumor cell destruction. Proc Natl Acad Sci U S A. (2011) 108:18342–7. doi: 10.1073/pnas.1106550108 PMC3215076 22042861

[64] Logtenberg MEW Jansen JHM Raaben M Toebes M Franke K Brandsma AM. Glutaminyl cyclase is an enzymatic modifier of the CD47- SIRPalpha axis and a target for cancer immunotherapy. Nat Med (2019) 25:612–9. doi: 10.1038/s41591-019-0356-z PMC7025889 30833751

[65] Zhao XW Kuijpers TW van den Berg TK. Is targeting of CD47-SIRPα enough for treating hematopoietic Malignancy? Blood (2012) 119:4333–4. doi: 10.1182/blood-2011-11-391367 22555661

[66] Chen J Zhong MC Guo H Davidson D Mishel S Lu Y. SLAMF7 is critical for phagocytosis of haematopoietic tumour cells *via* Mac-1 integrin. Nature (2017) 544:493–7. doi: 10.1038/nature22076 PMC5565268 28424516

[67] Mlecnik B Tosolini M Kirilovsky A Berger A Bindea G Meatchi T. Histopathologic-based prognostic factors of colorectal cancers are associated with the state of the local immune reaction. J Clin Oncol (2011) 29:610–8. doi: 10.1200/jco.2010.30.5425 21245428

[68] Galon J Mlecnik B Bindea G Angell HK Berger A Lagorce C. Towards the introduction of the 'Immunoscore' in the classification of Malignant tumours. J Pathol (2014) 232:199–209. doi: 10.1002/path.4287 24122236 PMC4255306

[69] Galon J Bruni D. Approaches to treat immune hot, altered and cold tumours with combination immunotherapies. Nat Rev Drug Discovery (2019) 18:197–218. doi: 10.1038/s41573-018-0007-y 30610226

[70] Quadrivalent vaccine against human papillomavirus to prevent high-grade cervical lesions. N Engl J Med (2007) 356:1915–27. doi: 10.1056/NEJMoa061741 17494925

[71] Kantoff PW Higano CS Shore ND Berger ER Small EJ Penson DF. Sipuleucel-T immunotherapy for castration-resistant prostate cancer. N Engl J Med (2010) 363:411–22. doi: 10.1056/NEJMoa1001294 20818862

[72] Harris JE Ryan L Hoover HC Jr. Stuart RK Oken MM Benson AB 3rd. Adjuvant active specific immunotherapy for stage II and III colon cancer with an autologous tumor cell vaccine: Eastern Cooperative Oncology Group Study E5283. J Clin Oncol (2000) 18:148–57. doi: 10.1200/jco.2000.18.1.148 10623705

[73] Chang AE Li Q Jiang G Sayre DM Braun TM Redman BG. Phase II trial of autologous tumor vaccination, anti-CD3-activated vaccine-primed lymphocytes, and interleukin-2 in stage IV renal cell cancer. J Clin Oncol (2003) 21:884–90. doi: 10.1200/jco.2003.08.023 12610189

[74] Jocham D Richter A Hoffmann L Iwig K Fahlenkamp D Zakrzewski G. Adjuvant autologous renal tumour cell vaccine and risk of tumour progression in patients with renal-cell carcinoma after radical nephrectomy: phase III, randomised controlled trial. Lancet (2004) 363:594–9. doi: 10.1016/s0140-6736(04)15590-6 14987883

[75] Biagi E Rousseau R Yvon E Schwartz M Dotti G Foster A. Responses to human CD40 ligand/human interleukin-2 autologous cell vaccine in patients with B-cell chronic lymphocytic leukemia. Clin Cancer Res (2005) 11:6916–23. doi: 10.1158/1078-0432.Ccr-05-0484 16203783

[76] Sanborn RE Ross HJ Aung S Acheson A Moudgil T Puri S. A pilot study of an autologous tumor-derived autophagosome vaccine with docetaxel in patients with stage IV non-small cell lung cancer. J Immunother Cancer (2017) 5:103. doi: 10.1186/s40425-017-0306-6 29258618 PMC5735525

[77] Frank MJ Khodadoust MS Czerwinski DK Haabeth OAW Chu MP Miklos DB. Autologous tumor cell vaccine induces antitumor T cell immune responses in patients with mantle cell lymphoma: A phase I/II trial. J Exp Med (2020), 217. doi: 10.1084/jem.20191712 PMC7478738 32558897

[78] Asada H Kishida T Hirai H Satoh E Ohashi S Takeuchi M. Significant antitumor effects obtained by autologous tumor cell vaccine engineered to secrete interleukin (IL)-12 and IL-18 by means of the EBV/lipoplex. Mol Ther (2002) 5:609–16. doi: 10.1006/mthe.2002.0587 11991752

[79] Dranoff G Jaffee E Lazenby A Golumbek P Levitsky H Brose K. Vaccination with irradiated tumor cells engineered to secrete murine granulocyte-macrophage colony-stimulating factor stimulates potent, specific, and long-lasting anti-tumor immunity. Proc Natl Acad Sci U S A. (1993) 90:3539–43. doi: 10.1073/pnas.90.8.3539 PMC46336 8097319

[80] Wu AA Bever KM Ho WJ Fertig EJ Niu N Zheng L. A phase II study of allogeneic GM-CSF-transfected pancreatic tumor vaccine (GVAX) with ipilimumab as maintenance treatment for metastatic pancreatic cancer. Clin Cancer Res (2020) 26:5129–39. doi: 10.1158/1078-0432.Ccr-20-1025 PMC7541669 32591464

[81] Ding Z Li Q Zhang R Xie L Shu Y Gao S. Personalized neoantigen pulsed dendritic cell vaccine for advanced lung cancer. Signal Transduct Target Ther (2021) 6:26. doi: 10.1038/s41392-020-00448-5 33473101 PMC7817684

[82] Butterfield LH. Cancer vaccines. Bmj (2015) 350:h988. doi: 10.1136/bmj.h988 25904595 PMC4707521

[83] Liu W Tang H Li L Wang X Yu Z Li J. Peptide-based therapeutic cancer vaccine: Current trends in clinical application. Cell Prolif (2021) 54:e13025. doi: 10.1111/cpr.13025 33754407 PMC8088465

[84] Steinman RM Banchereau J. Taking dendritic cells into medicine. Nature (2007) 449:419–26. doi: 10.1038/nature06175 17898760

[85] Sahin U Derhovanessian E Miller M Kloke BP Simon P Lower M. Personalized RNA mutanome vaccines mobilize poly-specific therapeutic immunity against cancer. Nature (2017) 547:222–6. doi: 10.1038/nature23003 28678784

[86] Lorentzen CL Haanen JB Met Ö Svane IM. Clinical advances and ongoing trials on mRNA vaccines for cancer treatment. Lancet Oncol (2022) 23:e450–8. doi: 10.1016/s1470-2045(22)00372-2 PMC9512276 36174631

[87] Daud AI DeConti RC Andrews S Urbas P Riker AI Sondak VK. Phase I trial of interleukin-12 plasmid electroporation in patients with metastatic melanoma. J Clin Oncol (2008) 26:5896–903. doi: 10.1200/jco.2007.15.6794 PMC2645111 19029422

[88] Aleksic M Liddy N Molloy PE Pumphrey N Vuidepot A Chang KM. Different affinity windows for virus and cancer-specific T-cell receptors: implications for therapeutic strategies. Eur J Immunol (2012) 42:3174–9. doi: 10.1002/eji.201242606 PMC3776049 22949370

[89] Cho JH Collins JJ Wong WW. Universal chimeric antigen receptors for multiplexed and logical control of T cell responses. Cell (2018) 173:1426–1438.e1411. doi: 10.1016/j.cell.2018.03.038 29706540 PMC5984158

[90] Urbanska K Lanitis E Poussin M Lynn RC Gavin BP Kelderman S. A universal strategy for adoptive immunotherapy of cancer through use of a novel T-cell antigen receptor. Cancer Res (2012) 72:1844–52. doi: 10.1158/0008-5472.Can-11-3890 PMC3319867 22315351

[91] Jayasooriya V Ringwelski B Dorsam G Nawarathna D. mRNA-based CAR T-cells manufactured by miniaturized two-step electroporation produce selective cytotoxicity toward target cancer cells. Lab Chip (2021) 21:3748–61. doi: 10.1039/d1lc00219h PMC8513750 34585697

[92] Valkenburg KC de Groot AE Pienta KJ. Targeting the tumour stroma to improve cancer therapy. Nat Rev Clin Oncol (2018) 15:366–81. doi: 10.1038/s41571-018-0007-1 PMC5960434 29651130

[93] Tran E Chinnasamy D Yu Z Morgan RA Lee CC Restifo NP. Immune targeting of fibroblast activation protein triggers recognition of multipotent bone marrow stromal cells and cachexia. J Exp Med (2013) 210:1125–35. doi: 10.1084/jem.20130110 PMC3674706 23712432

[94] Norelli M Camisa B Barbiera G Falcone L Purevdorj A Genua M. Monocyte-derived IL-1 and IL-6 are differentially required for cytokine-release syndrome and neurotoxicity due to CAR T cells. Nat Med (2018) 24:739–48. doi: 10.1038/s41591-018-0036-4 29808007

[95] Lee DW Santomasso BD Locke FL Ghobadi A Turtle CJ Brudno JN. ASTCT consensus grading for cytokine release syndrome and neurologic toxicity associated with immune effector cells. Biol Blood Marrow Transplant (2019) 25:625–38. doi: 10.1016/j.bbmt.2018.12.758 PMC12180426 30592986

[96] Allen GM Frankel NW Reddy NR Bhargava HK Yoshida MA Stark SR. Synthetic cytokine circuits that drive T cells into immune-excluded tumors. Science (2022) 378:eaba1624. doi: 10.1126/science.aba1624 36520915 PMC9970000

[97] Liu E Marin D Banerjee P Macapinlac HA Thompson P Basar R. Use of CAR-transduced natural killer cells in CD19-positive lymphoid tumors. N Engl J Med (2020) 382:545–53. doi: 10.1056/NEJMoa1910607 PMC7101242 32023374

[98] Liu E Tong Y Dotti G Shaim H Savoldo B Mukherjee M. Cord blood NK cells engineered to express IL-15 and a CD19-targeted CAR show long-term persistence and potent antitumor activity. Leukemia (2018) 32:520–31. doi: 10.1038/leu.2017.226 PMC6063081 28725044

[99] Gang M Marin ND Wong P Neal CC Marsala L Foster M. CAR-modified memory-like NK cells exhibit potent responses to NK-resistant lymphomas. Blood (2020) 136:2308–18. doi: 10.1182/blood.2020006619 PMC7702478 32614951

[100] Mantovani A Sozzani S Locati M Allavena P Sica A. Macrophage polarization: tumor-associated macrophages as a paradigm for polarized M2 mononuclear phagocytes. Trends Immunol (2002) 23:549–55. doi: 10.1016/s1471-4906(02)02302-5 12401408

[101] Nishiga Y Drainas AP Baron M Bhattacharya D Barkal AA Ahrari Y. Radiotherapy in combination with CD47 blockade elicits a macrophage-mediated abscopal effect. Nat Cancer (2022) 3:1351–66. doi: 10.1038/s43018-022-00456-0 PMC9701141 36411318

[102] Liu M Liu J Liang Z Dai K Gan J Wang Q. CAR-macrophages and CAR-T cells synergistically kill tumor cells. In Vitro Cells (2022) 11. doi: 10.3390/cells11223692 PMC9688246 36429120

[103] Morrissey MA Williamson AP Steinbach AM Roberts EW Kern N Headley MB. Chimeric antigen receptors that trigger phagocytosis. Elife (2018) 7. doi: 10.7554/eLife.36688 PMC6008046 29862966

[104] Zhang W Liu L Su H Liu Q Shen J Dai H. Chimeric antigen receptor macrophage therapy for breast tumours mediated by targeting the tumour extracellular matrix. Br J Cancer (2019) 121:837–45. doi: 10.1038/s41416-019-0578-3 PMC6889154 31570753

[105] Zhang L Tian L Dai X Yu H Wang J Lei A. Pluripotent stem cell-derived CAR-macrophage cells with antigen-dependent anti-cancer cell functions. J Hematol Oncol (2020) 13:153. doi: 10.1186/s13045-020-00983-2 33176869 PMC7656711

[106] Klichinsky M Ruella M Shestova O Lu XM Best A Zeeman M. Human chimeric antigen receptor macrophages for cancer immunotherapy. Nat Biotechnol (2020) 38:947–53. doi: 10.1038/s41587-020-0462-y PMC7883632 32361713

[107] Kaufman HL Kohlhapp FJ Zloza A. Oncolytic viruses: a new class of immunotherapy drugs. Nat Rev Drug Discovery (2015) 14:642–62. doi: 10.1038/nrd4663 PMC7097180 26323545

[108] Hietanen E Koivu MKA Susi P. Cytolytic properties and genome analysis of rigvir(®) oncolytic virotherapy virus and other echovirus 7 isolates. Viruses (2022) 14. doi: 10.3390/v14030525 PMC8949920 35336934

[109] Alberts P Tilgase A Rasa A Bandere K Venskus D. The advent of oncolytic virotherapy in oncology: The Rigvir® story. Eur J Pharmacol (2018) 837:117–26. doi: 10.1016/j.ejphar.2018.08.042 30179611

[110] Bommareddy PK Patel A Hossain S Kaufman HL. Talimogene laherparepvec (T-VEC) and other oncolytic viruses for the treatment of melanoma. Am J Clin Dermatol (2017) 18:1–15. doi: 10.1007/s40257-016-0238-9 27988837 PMC8977104

[111] Andtbacka RH Kaufman HL Collichio F Amatruda T Senzer N Chesney J. Talimogene laherparepvec improves durable response rate in patients with advanced melanoma. J Clin Oncol (2015) 33:2780–8. doi: 10.1200/jco.2014.58.3377 26014293

[112] Heise C Sampson-Johannes A Williams A McCormick F Von Hoff DD Kirn DH. ONYX-015, an E1B gene-attenuated adenovirus, causes tumor-specific cytolysis and antitumoral efficacy that can be augmented by standard chemotherapeutic agents. Nat Med (1997) 3:639–45. doi: 10.1038/nm0697-639 9176490

[113] Chen GX Zhang S He XH Liu SY Ma C Zou XP. Clinical utility of recombinant adenoviral human p53 gene therapy: current perspectives. Onco Targets Ther (2014) 7:1901–9. doi: 10.2147/ott.S50483 PMC4211860 25364261

[114] Wei D Xu J Liu XY Chen ZN Bian H. Fighting cancer with viruses: oncolytic virus therapy in China. Hum Gene Ther (2018) 29:151–9. doi: 10.1089/hum.2017.212 29284308

[115] Huang C Ren S Chen Y Liu A Wu Q Jiang T. PD-L1 methylation restricts PD-L1/PD-1 interactions to control cancer immune surveillance. Sci Adv (2023) 9:eade4186. doi: 10.1126/sciadv.ade4186 37235656 PMC10219601

